# Improved iterative shrinkage-thresholding for sparse signal recovery via Laplace mixtures models

**DOI:** 10.1186/s13634-018-0565-5

**Published:** 2018-07-13

**Authors:** Chiara Ravazzi, Enrico Magli

**Affiliations:** 1 National Research Council of Italy, IEIIT-CNR, c/o Politecnico di Torino, Corso Duca degli Abruzzi 24, Torino, 10129 Italy; 2 Politecnico di Torino, DET, Corso Duca degli Abruzzi 24, Torino, 10129 Italy

**Keywords:** Compressed sensing, Sparse recovery, Gaussian mixture models, MAP estimation, Mixture models, Reweighted *ℓ*_1_ minimization

## Abstract

In this paper, we propose a new method for support detection and estimation of sparse and approximately sparse signals from compressed measurements. Using a double Laplace mixture model as the parametric representation of the signal coefficients, the problem is formulated as a weighted *ℓ*_1_ minimization. Then, we introduce a new family of iterative shrinkage-thresholding algorithms based on double Laplace mixture models. They preserve the computational simplicity of classical ones and improve iterative estimation by incorporating soft support detection. In particular, at each iteration, by learning the components that are likely to be nonzero from the current MAP signal estimate, the shrinkage-thresholding step is adaptively tuned and optimized. Unlike other adaptive methods, we are able to prove, under suitable conditions, the convergence of the proposed methods to a local minimum of the weighted *ℓ*_1_ minimization. Moreover, we also provide an upper bound on the reconstruction error. Finally, we show through numerical experiments that the proposed methods outperform classical shrinkage-thresholding in terms of rate of convergence, accuracy, and of sparsity-undersampling trade-off.

## Introduction

In this paper, we consider the standard compressed sensing (CS) setting [1], where we are interested in recovering high-dimensional signals $x^{\star }\in \mathbb {R}^{n}$ from few linear measurements *y*=*Ax*^⋆^+*η*, where $A\in \mathbb {R}^{m\times n}$, *m*≪*n*, and *η* is a Gaussian i.i.d. noise. The problem is underdetermined and has infinitely many solutions. However, much interest has been focused on finding the sparsest solution, i.e., the one with the smallest number of nonzero components [2]. This involves the minimization of *ℓ*_0_ pseudonorm [3], which is NP-hard.

A practical alternative is to use the *ℓ*_1_ regularization, leading to the basis pursuit (BP, [4]) problem in the absence of noise, or the least absolute shrinkage and selection operator (Lasso, [5]) in the presence of noise. They can be efficiently solved by iterative shrinkage-thresholding algorithms (ISTA, [6–8]) that are generally first-order methods followed by a shrinkage-thresholding step. Due to its implementation simplicity and suitability for high-dimensional problems, a large effort has been spent to improve their speed of convergence [9–12], asymptotic performance in the large system limit [13, 14], and ease of use [15].

In a Bayesian framework, *ℓ*_1_ minimization is equivalent to a maximum a posteriori (MAP) estimate [16] modeling the signal coefficients using a Laplace prior, in the sense that we need to solve the same optimization problem. Although the Laplace probability density function does not provide a relevant generative model for sparse or compressible signals [17], the non-differentiability at zero of the cost function leads to select a sparse solution, providing empirical success of *ℓ*_1_ regularization.

However, *ℓ*_1_ minimization alone does not fully exploit signal sparsity. In fact, in some cases, a support estimate [18] could be employed to reduce the number of measurements needed for good reconstruction via BP or Lasso, e.g., by combining support detection with weighted or truncated *ℓ*_1_ minimization [19]. The idea of combining support information and signal estimation has appeared in CS literature with several assumptions [20–27]. For example, in [25], the authors employ as prior information an estimate *T* of the support of the signal and propose a truncated *ℓ*_1_ minimization problem. Another piece of literature [28] considers a weighted *ℓ*_1_ minimization with weights *w*_*i*_=− log*p*_*i*_ where *p*_*i*_ is the probability that $x^{\star }_{i} = 0$.

In this paper, we propose an iterative soft support detection and estimation method for CS. It is worth remarking that in our setting prior information on the support *T* or *p*_*i*_ is not available. The fundamental idea is to combine the *good geometric properties of the* *ℓ*_1_*cost function* associated to the Laplacian prior with a *good generative model* for sparse and compressible vectors [17]. For this purpose, we use a Laplace mixture model as the parametric representation of the prior distribution of the signal coefficients. Because of the partial symmetry of the signal sparsity, we know that each coefficient should have one out of only two distributions: a Laplace with small variance with high probability and a Laplace with large variance with low probability. We show empirically that this model fits better with the distribution of the Haar wavelet coefficients in test images. Then, we cast the estimation problem as a weighted *ℓ*_1_ minimization method that incorporates the parametric representation of the signal.

We show that the proposed framework is able to improve a number of existing methods based on shrinkage-thresholding: by estimating the distribution of the components that are likely to be nonzero from signal estimates at each iteration (support detection), the shrinkage-thresholding step is tuned and optimized, thereby yielding better estimation. As opposed to other adaptive methods [10], we are able to prove, under suitable conditions, the convergence of the proposed tuned method. Moreover, we derive an upper bound on the reconstruction error. We apply this method to several algorithms, showing by numerical simulation that it improves recovery in terms of both speed of convergence and sparsity-undersampling trade-off, while preserving the implementation simplicity.

Compared to the literature on reconstruction methods that combine iterative support detection and weighted *ℓ*_1_ minimization, the identification of the support is not nested or incremental over time as in [29–31]. Moreover, the choice of weights in *ℓ*_1_ minimization is based on the Bayesian rules and a probabilistic model and not on greedy rules as in [19, 32]. This feature also marks the difference with respect to reweighted *ℓ*_1_/ *ℓ*_2_ minimization, where the weights are chosen with the aim of approximating the *ℓ*_*τ*_ norm with *τ*∈(0,1].

### Outline

The paper is organized as follows. In Section 2, the basic CS theory and the classical methods based on *ℓ*_1_ minimization for sparse recovery are reviewed. The proposed parametric model for sparse or highly compressible signals is described in Section 3 and compared with the related literature. In Section 4, the estimation problem based on Laplace mixture models is introduced and recast as a weighted *ℓ*_1_ minimization problem. Then, in Section 5, the proposed approach is used to improve a number of existing methods based on shrinkage-thresholding. Numerical experiments are presented in Section 6 and some concluding remarks (Section 7) complete the paper. The theoretical results are rigorously proved in Appendices 1, 2, 3, and 4.

### Notation

We conclude this introduction with some notation. We denote column vectors with small letters and matrices with capital letters. If $x\in \mathbb {R}^{n}$, we denote its *j*th element with *x*_*j*_ and, given *S*∈[*n*]:={1,…,*n*}, by *x*|_*S*_ the subvector of *x* corresponding to the indexes in *S*. The support set of *x* is defined by supp(*x*)={*i*∈[*n*]:*x*_*i*_≠0}, and we use ∥*x*∥_0_=|supp(*x*)|. Finally, the symbol ∥*x*∥ with no subscript is to be understood as the Euclidean norm of the vector *x*. We denote as *r*(*x*) the nonincreasing rearrangement of *x*

$$r(x)=\left(|x_{i_{1}}|,|x_{i_{2}}|,\ldots,|x_{i_{n}}|\right)^{\top}, $$

where

$$|x_{i_{\ell}}|\geq|x_{i_{\ell+1}}|,\ \forall\ell=1,\ldots,n-1. $$

We denote with $\Sigma _{s}=\{x\in \mathbb {R}^{n}:|\text {supp}(x)|\leq s\}$ and define

$$\sigma_{s}(x)=\underset{z\in\Sigma_{s}}{\text{arg\ min}}\|x-z\|. $$

It should be checked that

$$\sigma_{s}(x)_{i}= \left\{\begin{array}{ll} x_{i}&\text{if }|x_{i}|> r(x)_{s+1}\\ 0&\text{otherwise}. \end{array}\right. $$

Given a matrix *A*, *A*^T^ denotes its transpose.

## Mathematical formulation

### Sparse signal recovery from compressed measurements

Compressive sensing aims to recover a sparse signal $x^{\star }\in \mathbb {R}^{n}$ from *m*≤*n* random projections of the form

1 $$ y=Ax^{\star}+\eta  $$

where $y\in \mathbb {R}^{m}$ is the observation vector, $A\in \mathbb {R}^{m\times n}$ is the measurement matrix, and *η* is an additive noise. For example, in the transform domain compressive signal reconstruction [1], *A*=*ΦΨ*, where $\Psi \in \mathbb {R}^{n\times n}$ is the sparsifying basis (i.e., multiplying by *Ψ* corresponds to performing inverse transform), the entries of *x*^⋆^ is the transform coefficient vector that has *k* nonzero entries, and $\Phi \in \mathbb {R}^{m\times n}$ is the sensing matrix, whose rows are incoherent with the columns of *Ψ*.

Conventional reconstruction methods involve *ℓ*_1_ regularization [4]. In particular, it has been shown that, in the absence of noise and under suitable assumptions on the matrix *A*, the basis pursuit problem (BP)

2 $$  \min_{x\in\mathbb{R}^{n}}\|x\|_{1}\qquad \text{s.t.} \quad Ax=y  $$

can exactly recover a *k*-sparse signal (i.e., with a number of nonzero coefficients not larger than *k*) from *m*=*O*(*k* log(*n*/*k*)) measurements with high probability [33, 34]. In the presence of noise, one of the most popular convex relaxation methods is the least absolute shrinkage and selection operator (Lasso, [5]), which requires to solve the following unconstrained problem

3 $$ \min_{x\in\mathbb{R}^{n}}\left[\lambda\|x\|_{1}+\frac{1}{2} \|Ax-y\|_{2}^{2}\right]  $$

where *λ* is a positive regularization parameter.

Even when the vector is not exactly sparse, under *compressibility* assumptions of the signals to be recovered, the *ℓ*_1_ regularization provides estimates with a controlled error [33]. More formally, we recall the following definition [17].

#### **Definition 1**

(Compressible vectors) A vector $x\in \mathbb {R}^{n}$ is compressible if, denoted $\varrho _{k}(x):=\inf _{\|z\|_{0}\leq k}\|z-x\|$, the relative best *k*-term approximation error is $\overline {\varrho }_{k}(x): ={\varrho _{k}(x)}/{\|x\|}<\!\!\!<1$ for some *k*< <*n*.

If $x\in \mathbb {R}^{n}$ is not exactly sparse but compressible, the support is intended as the set of significant components supp(*ϱ*_*k*_(*x*)). One drawback of classical *ℓ*_1_ minimization is that it fails to penalize the coefficients in different ways. In this paper, we propose a new family of methods that incorporate two tasks: iterative support detection and signal recovery.

## Discussion: learning sparsity models

### A Bayesian view

In a Bayesian framework, if the noise in (1) is white Gaussian (we suppose for simplicity unitary standard deviation), BP and Lasso may be interpreted as a Bayesian MAP estimate [16]. In fact, imposing the *ℓ*_1_ norm as penalty in the cost function is equivalent to modeling the signal coefficients $x^{\star }_{i}$ as independent and identically distributed as a Laplace distribution, namely

$$\widehat{x}^{\ast}(y)=\underset{x\in\mathbb{R}^{n}}{\text{arg\ max}}\log f^{\ast}_{x|y}(x|y) $$

where, using the Bayes rule, *f*_*x*|*y*_(*x*|*y*) is given by

$$f^{\text{BP}}_{x|y}(x|y)= \frac{1}{Z}\prod_{i=1}^{n}\exp({-\lambda |x_{i}|})\prod_{j=1}^{m}\delta_{y_{j}=(Ax)_{j}}, $$

in case of BP, and for Lasso by

$$\begin{aligned} f_{x|y}^{\text{Lasso}}(x|y)&= \frac{1}{Z}\prod_{i=1}^{n}\exp({-\lambda |x_{i}|})\\ &\quad\times\prod_{j=1}^{m}\exp\left(-\frac{1}{2}\left(y_{j}-(Ax)_{j}\right)^{2}\right), \end{aligned} $$

and *Z* is a normalization factor so that $\int f^{\ast }_{x|y}(x|y)\mathrm {d}x=1$.

Despite the good geometric properties of the *ℓ*_1_ cost function associated to such prior, that allow to select a sparse solution, the Laplace prior does not provide a relevant generative model for sparse or compressible signals [17]. In fact, if $x_{n}\in \mathbb {R}^{n}$ is distributed as i.i.d. with respect to Laplace distribution with scale parameter *λ*, then for any sequence *k*_*n*_ such that ${\lim }_{n\rightarrow \infty }k_{n}/n=\kappa \in [0,1]$, it holds almost surely

$$ \varepsilon:={\lim}_{n\rightarrow\infty}\overline{\varrho}_{k_{n}}(x_{n})^{2}\overset{a.s.}{=}1 - \kappa\left(1+\log1/\kappa+\frac{1}{2}(\log1/\kappa)^{2}\right)\!. $$

Therefore, the vectors generated from i.i.d. Laplace distribution are not compressible since we cannot have both *κ* and *ε* small at the same time.

**Fig. 1:**
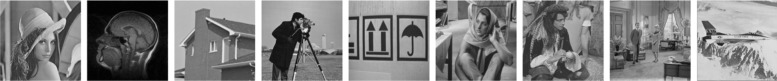
Test images for experiments 1 and 2: Lena, MRI-head, house, cameraman, pattern, Barbara, man, couple, plane (from left to right)

**Fig. 2:**
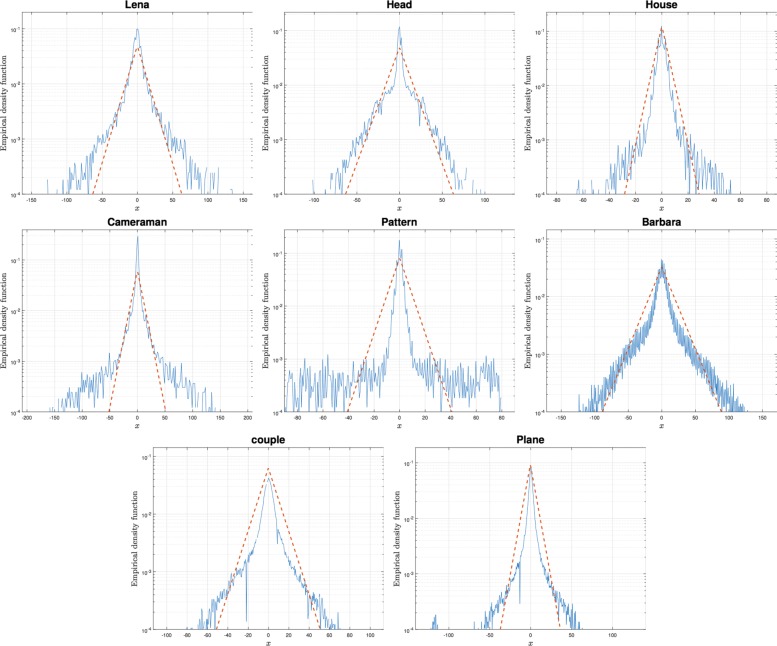
Experiment 1: empirical density function in the log domain of a single vertical wavelet subband coefficients of several real images (solid) and the best fitting of the Laplace density function (dashed) obtained by maximizing the likelihood of the data

Moreover, for a large class of real signals that have highly non-Gaussian statistics, the Laplace model does not provide a good fit to the empirical probability density function. We show this with a simple experiment (experiment 1) Fig. 1. We calculate a single vertical wavelet subband coefficients of several real images of size 256 × 256 pixels, and we compute for each image the best fitting of the double Laplace density function obtained by maximizing the likelihood of the data under that assumption. In Fig. 2, the empirical density function of the Haar wavelet coefficients using 256 bins is shown in the log domain (solid line) and the dashed line corresponds to the best fitting instance of the Laplace density function. It should be noticed that the Laplace density captures peaks at zero but is less accurate along the tails.

In [35], Lasso is proved to provide a robust estimation that is invariant to the signal prior. In sharp contrast, the Bayesian Lasso is able to provide an estimator with minimum mean squared error by incorporating the signal model in the estimation problem [14, 36], but the assumption that the signal prior is known in advance is not reasonable in most practical cases. Hence, it becomes crucial to incorporate in the recovery procedure new tools for adaptively learning sparsity models. Other models have been proposed for compressible signals [37–39] using more accurate probability density functions than double Laplace distribution. However, two issues generally appear when an accurate but complex signal prior is used: (1) it can be hard to estimate the model parameters and (2) the optimal estimators may not have simple closed form solution and their computation may require high computational work [40]. In fact, although the double Laplace prior is not the most accurate model, this is an especially convenient assumption since the MAP estimator has a simple and closed form [41].

Our goal is to use a compressible distribution as parametric representation of the signal coefficients, able to combine support detection and estimation, and to preserve the simplicity and advantages coming from the Laplace prior assumption.

### Proposed approach: two-component Laplace mixture for support detection

We consider a two-state mixture model as a prior that describes our knowledge about the sparsity of the solution to (1). Because of the partial symmetry of the signal sparsity, we consider the case in which *x* is a random variable with components of the form

$$x_{i}={z_{i}}u_{i}+{(1-z_{i})}v_{i}\quad i\in[n] $$

where *u*_*i*_ are identically and independently distributed (i.i.d.) as *Laplace*(0,*α*), *v*_*i*_ are i.i.d. according to *Laplace*(0,*β*) and *z*_*i*_ are i.i.d. Bernoulli random variables with probability mass function *f*(*z*_*i*_=1)=1−*p*, with *p*< <1/2, *α*≈0, and *β*> >0, in order to ensure that we have few large coefficients. We thus consider the conditional distribution of the data: let *Θ*=(*α*,*β*)

4 $$   \begin{aligned} f(x|y;\Theta) &= \frac{1}{Z}\prod_{i=1}^{n}\left[(1-p)f(x_{i}|z_{i}=1)+p f(x_{i}|z_{i}=0)\right]\\ &\quad\times\prod_{j=1}^{m} f_{j}(y|x), \end{aligned}  $$

where

5a $$ f(x_{i}|z_{i}=1)=\frac{1}{2\alpha}\exp\left(-\frac{|x_{i}|}{\alpha}\right)  $$

5b $$ f(x_{i}|z_{i}=0)=\frac{1}{2\beta}\exp\left(-\frac{|x_{i}|}{\beta}\right),  $$

$$f_{j}(y|x)=\delta_{\{y_{j}=(Ax)_{j}\}} $$

in absence of noise or

$$f_{j}(y|x)=\frac{1}{\sqrt{2\pi}}\exp\left(-\frac{1}{2}\left(y_{j}-(Ax)_{j}\right)^{2}\right) $$

in presence of noise, and *Z* is a normalization factor so that $\int f(x|y;\Theta)\mathrm {d}x=1$.

This mixture model is completely described by three parameters: the sparsity ratio *p*< <1/2, *α* that is expected to be small, and *β*>*α* if the signal is sparse. It should be noticed that vectors generated from this distribution are typically compressible according to Definition 1.

#### **Proposition 1**

Let $x_{n}\in \mathbb {R}^{n}$ be i.i.d. with respect to (4). Then, for any sequence *k*_*n*_ such that ${\lim }_{n\rightarrow \infty }k_{n}/n=\kappa \in [0,1]$, it holds almost surely

$${{ \begin{aligned} \varepsilon&:={\lim}_{n\rightarrow\infty}\overline{\varrho}_{k_{n}}(x_{n})^{2}\\ &\overset{a.s.}{=}\!\frac{(1\,-\,p)\!\left(\alpha^{2}\,-\,\mathrm{e}^{-t/\alpha}\left(t^{2}/2\,+\,\alpha t\,+\,\alpha^{2}\right)\right)\,+\,p\!\left(\beta^{2}\,-\,\mathrm{e}^{-t/\beta}\left(t^{2}/2\,+\,\beta t\,+\,\beta^{2}\right)\!\right)}{(1\,-\,p)\alpha^{2}\,+\,p\beta^{2}}\\ \end{aligned}}} $$

where *t* is the unique solution of

$$(1-p)\mathrm{e}^{-t/\alpha}+p\mathrm{e}^{-t/\beta}=\kappa. $$

The proof is a consequence of proposition 1 in [17] and is deferred to Appendix 1.

**Fig. 3:**
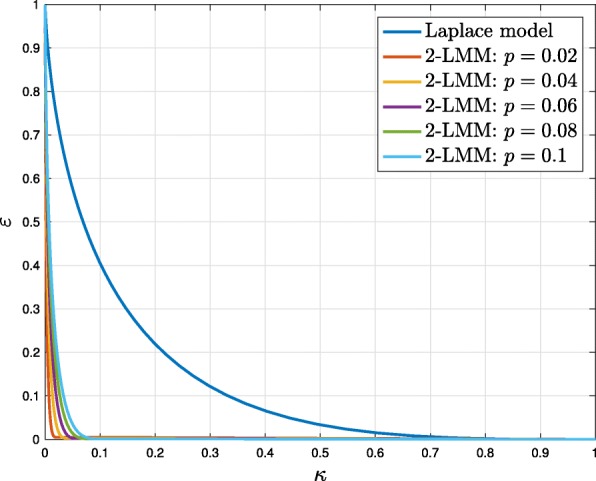
Experiment 2: comparison of compressibility parameters (*κ*,*ε*) for several priors. Laplace distribution is not a compressible distribution, whereas for 2-LMM distribution if parameter *p* is small and *α*< <*β* we can have both *κ* and *ε* small at the same time. In this case *α*=0.1, *β*=10, and *p*∈[0.02,0.1]

In Fig. 3 (experiment 2), we compare the compressibility parameters (*κ*,*ε*) of the Laplace distribution and of 2-LMM distribution with *α*=0.1,*β*=10 for several values of *p*. It should be noted that Laplace distribution is not a compressible distribution (we can not have *κ* and *ε* small at the same time), whereas 2-LMM distribution are compressible if parameter *p* is sufficiently small, as we can have both *κ* and *ε* small at the same time.

**Fig. 4:**
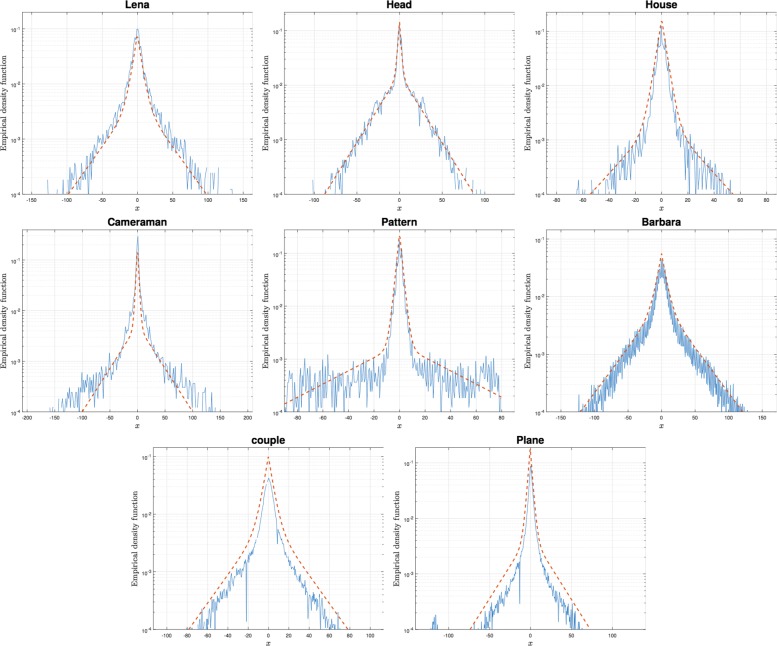
Experiment 2: empirical density function in the log domain of a single vertical wavelet subband coefficients of natural images (solid). The dashed line represents the best fitting of the mixture of two Laplace density functions computed by maximizing the likelihood of the data

We now compute the empirical probability density function of the Haar wavelet coefficients of several images and the best fitting of the mixture of two Laplace density functions computed by maximizing the likelihood of the data. The computation has been carried out via expectation maximization algorithm [16]. In Fig. 4, we show the results for several images. In order to compare the two parametric representations of sparsity, in Table 1, the Kullback-Leibler divergence of the best fitting probability models and the empirical probability density function are computed for the two models. It can be noticed that a single Laplace is a poor model for the Haar wavelet coefficients of natural images. The better accuracy obtained by the new parametric representation is evident.

**Table 1:** The Kullback-Leibler divergence of the best fitting probability models and the empirical probability density function are computed for the two models

Image	Lena	MRI-head	House	Cameraman	Pattern	Barbara	Man	Couple	Plane
Lap	0.1266	0.2109	0.1593	0.4958	0.6756	0.1449	0.0941	0.1793	0.4044
2-LMM	0.0688	0.0339	0.0688	0.0435	0.0818	0.0651	0.0322	0.0558	0.0906

## Method: support detection and sparse signal estimation via the 2-LMM

### Estimation using the 2-LMM generative model

Let us consider the logarithm of the conditional distribution in (4) in absence of noise (although similar consideration can be done in presence of noise):

6 $$ L(x;\Theta):=\log\left[f(x|y;\Theta)\right]  $$

For convenience, we consider *p* fixed as a guess of the degree of signal’s sparsity, whereas *Θ*=(*α*,*β*) will be unknown. The choice to keep *p* fixed does not entail a significant restriction to our analysis.

#### **Proposition 2**

The following optimization problems are equivalent

7 $$\begin{array}{*{20}l} &\max_{\Theta}\max_{x\in\mathbb{R}^{n}}L(x;\Theta) \end{array} $$

8 $$\begin{array}{*{20}l} &\min_{\Theta}\min_{x\in\mathbb{R}^{n}}\min_{\pi\in [0,1]^{n}}\underbrace{J(x,\pi;\Theta)-\sum_{i=1}^{n}H(\pi_{i})}_{V(x,\pi;\Theta)}\quad \text{s.t.} Ax=y \end{array} $$

where

9 $$\begin{array}{*{20}l} {J}(x,{\pi};\Theta)&=\sum_{i=1}^{n}\left[\frac{{\pi}_{i}|x_{i}|}{\alpha}+{{\pi}_{i}}\log\alpha-\pi_{i}\log(1-p)\right.\\ &\quad+\!\left.\frac{(1\,-\,{\pi}_{i})|x_{i}|}{\beta}\,+\,{(1\,-\,{\pi}_{i})}\log\beta\,-\,(1\,-\,{\pi}_{i})\log p\right], \end{array} $$

and *H*(*t*)=−*t* log*t*−(1−*t*) log(1−*t*)is the natural entropy function with *t*∈[0,1].

Given *y*,*A*, instead of solving optimization problem in (7), we consider the minimization of the following modified cost function:

10 $$\begin{array}{*{20}l} \min_{\Theta}\min_{x\in\mathbb{R}^{n}} \min_{\pi\in\Sigma_{n-K}} {J_{\epsilon}(x,\pi;\Theta)-\sum_{i=1}^{n}H(\pi_{i})}, \text{s.t.} Ax=y \end{array} $$

where *K*=⌊*pn*⌋, and

11 $$\begin{array}{*{20}l}  {J}_{\epsilon}(x,\pi;\Theta)&:=J(x,\pi;\Theta)+\epsilon\left(\frac{1}{\alpha}+\frac{1}{\beta}\right)\\ &=\sum_{i=1}^{n}\left[\frac{\pi_{i}|x_{i}|+\epsilon/n}{\alpha}+{\pi_{i}}\log\alpha-\pi_{i}\log(1-p)\right.\\ &\quad+\frac{(1-\pi_{i})|x_{i}|+\epsilon/n}{\beta}+{(1-\pi_{i})}\log\beta\\ &\quad-\left.\vphantom{\frac{(1-\pi_{i})|x_{i}|+\epsilon/n}{\beta}}(1-\pi_{i})\log p\right] \end{array} $$

Compared to (7), the optimization problem in (10)

1. Introduces the *ε* parameter, which is a regularization term used to avoid singularities whenever one of the Laplace components collapses onto a specific data point since we expect that *α*≈0, since we seek a sparse solution; this fact will be clear later;
2. Introduces the constraint *π*∈*Σ*_*n*−*K*_, which enforces a sparse solution.

Similar computations can be carried out for the case with noise, leading to

12 $$  \begin{aligned} \min_{\Theta}\min_{x\in\mathbb{R}^{n}}& \min_{\pi\in\Sigma_{n-K}}V(x,\pi,\alpha,\beta,\epsilon)\\ V(x,\pi,\alpha,\beta,\epsilon):&=\frac{1}{2}\|y-Ax\|_{2}^{2}\! +\!\lambda J_{\epsilon}(x,\pi;\Theta)\,-\,\lambda\!\sum_{i=1}^{n}H(\pi_{i}) \end{aligned}  $$

It should be noted that there is not a closed form solution to problems (10) and (12).

However, partial minimizations of *V*_*ε*_=*V*(*x*,*π*,*α*,*β*,*ε*) with respect to just one of the variables have simple representation. More precisely, we have the following expressions (see Lemma 3 in Appendix 3).

#### **Proposition 3**

Let

$$\begin{array}{*{20}l} \widehat{\pi}&=\widehat{\pi}(x,\alpha,\beta,\epsilon)=\underset{\xi\in\Sigma_{n-K}}{\mathrm{arg\ min}} V_{\epsilon}(x,\xi,\alpha,\beta)\\ \widehat{\alpha}&=\widehat{\alpha}(x,\alpha,\beta,\epsilon)=\underset{\alpha\in\mathbb{R}}{\mathrm{arg\ min}} V_{\epsilon}(x,\xi,\alpha,\beta)\\ \widehat{\beta}&=\widehat{\beta}(x,\alpha,\beta,\epsilon)=\underset{\beta\in\mathbb{R}}{\mathrm{arg\ min}} V_{\epsilon}(x,\xi,\alpha,\beta) \end{array} $$

then

13 $$\begin{array}{*{20}l} \widehat{\pi}& = \sigma_{n-K}\left(\frac{\mathrm{e}^{-\frac{|x|}{\alpha}-{\log(\alpha)}+\log(1-p)}}{\mathrm{e}^{-\frac{|x|}{\alpha}-\log(\alpha)+\log(1-p)} + \mathrm{e}^{-\frac{|x|}{\beta}-{\log(\beta)}+\log p}}\right) \end{array} $$

and

$$\widehat{\alpha}=\frac{\sum_{i=1}^{n}\pi_{i}|x_{i}|+\epsilon}{\sum_{j=1}^{n}\pi_{j}},\quad \widehat{\beta}=\frac{\sum_{i=1}^{n}(1-\pi_{i})|x_{i}|+\epsilon}{\sum_{j=1}^{n}(1-\pi_{j})} $$

In the following section, we present several iterative algorithms to approximately solve these optimization problems.

## Proposed iterative methods and main results

### Iterative shrinkage/thresholding algorithms

The literature describes a large number of approaches to address minimization of (2) and (3). Popular iterative methods belong to the class of iterative shrinkage-thresholding algorithms. These methods can be understood as a special proximal forward backward iterative scheme [42] and are appealing as they have lower computational complexity per iteration and lower storage requirements than interior-point methods. In fact, these types of recursions are a modification of the gradient method to solve a least square problem, where the dominant computational effort lies in a relatively cheap matrix-vector multiplication involving *A* and *A*^⊤^ and the only difference consists in the application of a shrinkage/soft thresholding operator, which promotes sparsity of the estimate at each iteration.

More precisely, let $\left \{\tau ^{(t)}\right \}_{t\in \mathbb {N}}$ be a sequence in (0,*∞*) such that $\inf _{t\in \mathbb {N}}\tau ^{(t)}>0$ and $\sup _{t\in \mathbb {N}}\tau ^{(t)}<2\|A\|_{2}^{-2}$, and let $\left \{u^{(t)}\right \}_{t\in \mathbb {N}}$ be a sequence in $\mathbb {R}^{n}$. Then, for every $t\in \mathbb {N}$ let

14 $$ x^{(t+1)}=\eta^{\mathsf{S}}_{\lambda\tau^{(t)}}\left[x^{(t)}+\tau^{(t)} A^{\mathsf{T}}\left(y-Ax^{(t)}\right){{+u^{(t)}}}\right],  $$

where $\eta ^{\mathsf {S}}_{\gamma }$ is a thresholding function to be applied element-wise defined as

15 $$ \eta^{\mathsf{S}}_{\gamma}\left[x\right]= \left\{\begin{array}{ll} \text{sgn}(x)(|x|-\gamma)&\text{if}\,|x|>\gamma\\ 0&\text{otherwise.} \end{array}\right.  $$

The simplest form, known as iterative shrinkage-thresholding algorithms (ISTA, [6]), considers *u*^(*t*)^=0 and $\tau ^{(t)}=\tau <2\|A\|_{2}^{-2}$ for all $t\in \mathbb {N}$. This algorithm is guaranteed to converge to a minimizer of the Lasso [6]. Moreover, as shown in [43], if *A* fulfills the so-called finite basis injectivity condition, the convergence is linear. However, the factor determining the speed within the class of linearly convergent algorithms depends on local well conditioning of the matrix *A*, meaning that ISTA can converge arbitrarily slowly in some sense, which is also often observed in practice.

In order to speed up ISTA, alternative algorithms have exploited preconditioning techniques or adaptivity, combining a decreasing thresholding strategy with adaptive descent parameter. However, the lack of a model-based thresholding policy makes this algorithm very sensitive to the signal statistics and the accuracy is not always guaranteed. In [13], the thresholding and descent parameters are optimally tuned in terms of phase transitions, i.e., they maximize the number of nonzeros at which the algorithm can successfully operate. However, preconditioning can be very expensive and there is no proof of convergence for adaptive methods.

Finally, other variations update the next iterate using not only the previous estimation, but two or more previously computed iterates. Among all the proposed techniques with a significantly better rate of convergence and phase transitions, we recall (a) fast iterative shrinkage-thresholding algorithm (FISTA, [9]) obtained by (14) choosing $\tau ^{(t)}=\tau <2\|A\|_{2}^{-2}$ and

16 $$ \begin{aligned} u^{(t)}&=\frac{\zeta^{(t-1)}-1}{\zeta^{(t)}}\left(I-\tau A^{\mathsf{T}}A\right)\left(x^{(t)}-x^{(t-1)}\right)\\ \zeta^{(0)}&=1,\quad \zeta^{(t+1)}=\frac{1+\sqrt{1+4\left(\zeta^{(t)}\right)^{2}}}{2} \end{aligned}  $$

and (b) approximate message passing (AMP, [14]) with threshold recursion proposed in [44]

17 $$ {\begin{aligned} u^{(t)}&={\left(1-\tau^{(t)}\right)}A^{\mathsf{T}}\left(Ax^{(t)}-y\right)+\frac{\left\|x^{(t)}\right\|_{0}}{m}A^{\mathsf{T}}r^{(t-1)}\\ \tau^{(t)}&=\chi \frac{\left\|r^{(t)}\right\|_{2}}{\sqrt{m}},\quad r^{(t)}=y-Ax^{(t)}+r^{(t-1)}. \end{aligned}}  $$

In this section, we show how to adapt these numerical methods to solve the weighted minimization problem via 2-LMM.

### 2-LMM-tuned iterative shrinkage-thresholding

Let us consider the problem of minimizing (11). Since information about the locations of the nonzero coefficients of the original signal is not available a priori, the task of selecting the parameters *α*,*β*, and *π* is performed iteratively. We propose an alternating method for the minimization of (11), inspired by the EM algorithm [16]. The pseudocode of the algorithm is reported in Algorithm 1. The strategy can be summarized as follows.

1. Let *t*:=0 and set an initial estimate *K* for the sparsity level, *p*=*K*/*n*, a small value *α*^(0)^≈0 (e.g., *α*^(0)^=0.1), the initial configuration , and *ε*^(0)^=1. Since *π*^(0)^=**1**, *β*^(0)^ can be arbitrary since it is not used in the first step of the algorithm.
2. Given the observed data *y* and current parameters *π*_*i*_,*α*,and*β*, a new estimation *x*^(*t*+1)^ of the signal is obtained by moving in a minimizing direction of weighted Lasso
  18 $$ F(x)= \frac{1}{2}\|Ax-y\|^{2}+\lambda\sum_{i=1}^{n}\omega_{i}^{(t+1)}|x_{i}|  $$
  with $\omega _{i}^{(t+1)}=\pi _{i}/\alpha +(1-\pi _{i})/\beta $; in other terms *x*^(*t*+1)^ is such that *F*(*x*^(*t*+1)^)≤*F*(*x*^(*t*)^).
3. The posterior distribution of the signal coefficients is evaluated and thresholded by keeping its *n*−*K* biggest elements and setting the others to zero. It is worth remarking that this step differs from the E-step of a classical EM algorithms as a thresholding operator *σ*_*n*−*K*_ is applied in order to promote the sparsity in the probability vector *π*.
4. Given the probabilities, we use them to re-estimate the mixture parameters *α*^(*t*)^ and *β*^(*t*)^.
5. Set *t*:=*t*+1 and iterate until the stopping criteria is satisfied, e.g., until the estimate stops changing ∥*x*^(*t*+1)^−*x*^(*t*)^∥/∥*x*^(*t*)^∥<*tol* for some *tol*>0.

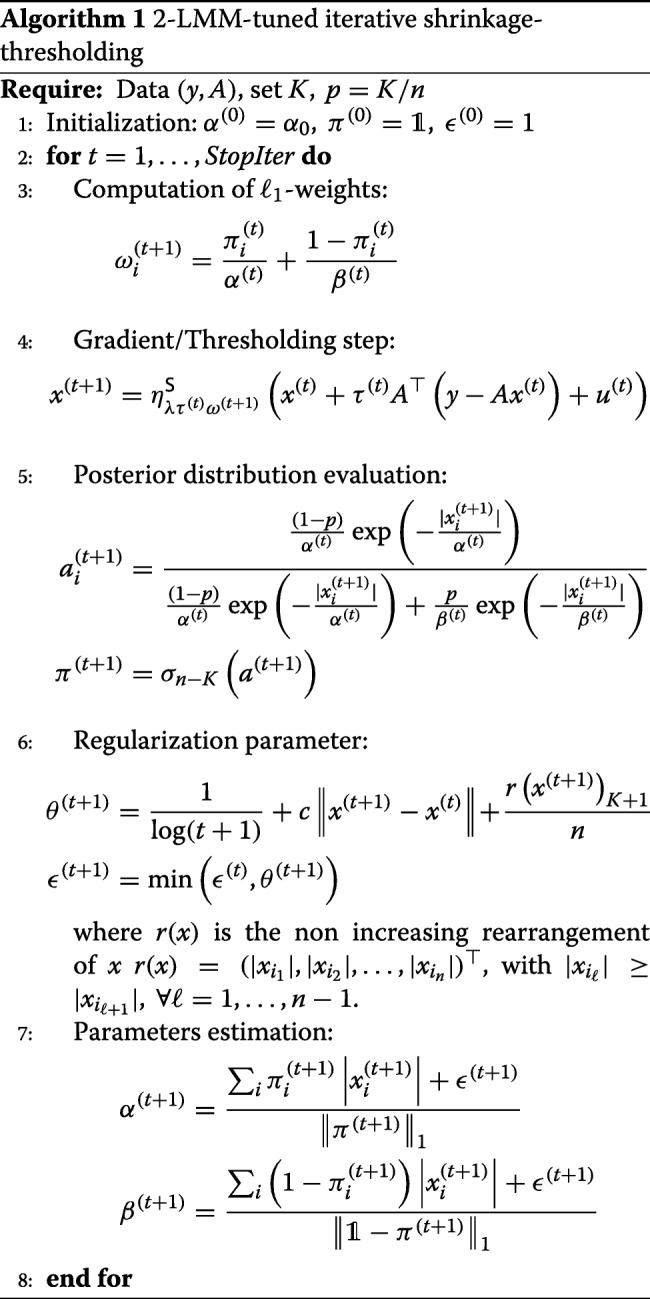


### Relation to prior literature

As already observed, Algorithm 1 belongs to the more general class of methods for weighted *ℓ*_1_ norm minimization [45–47] (see (18)). Common strategies for iterative reweighting *ℓ*_1_ minimization (IRL1, [45]) that have been explored in literature re-compute weights at every iteration using the estimate at the previous iteration ${\omega _{i}^{(t+1)}=\chi /(|x^{(t)}_{i}|+\epsilon)}$ where *χ* and *ε* are appropriate positive constants. In Algorithm 1, the weights $\omega _{i}^{(t)}$ are chosen to jointly fit the signal prior and, consequently, depend on all components of the signal and not exclusively on the value $x^{(t)}_{i}$. Our strategy is also related to threshold-ISD [19] that incorporates support detection in the weighted *ℓ*_1_ minimization and runs as fast as the basis pursuit. Given a support estimate, the estimation is performed by solving a truncated basis pursuit problem. Also in [48], an iterative algorithm, called WSPGL1, is designed to solve a sequence of weighted LASSO using a support estimate, derived from the data, and updated at every iteration. Compared to threshold-ISD and WSPGL1, 2-LMM-tuned iterative shrinkage-thresholding does not use binary weights and is more flexible. Moreover, in threshold-ISD, like CoSaMP, the identification of the support is based on greedy rules and not chosen to optimally fit the prior distribution of the signal.

A prior estimation based on EM was incorporated within the AMP framework also in [49] where a Gaussian mixture model is used as the parametric representation of the signal. The key difference in our approach is that model fitting is used to estimate the support and to adaptively select the best thresholding function with the minimum mean square error. The necessity of selecting the best thresholding function is also proposed in parametric SURE AMP [50] where a class of parametric denoising functions is used to adaptively choose the best-in-class denoiser. However, at each iteration, parametric SURE AMP needs to solve a linear system and the number of parameters affects heavily both performance and complexity.

### Convergence analysis

Under suitable conditions, we are able to guarantee the convergence of the iterates produced by Algorithm I and discuss sufficient condition for optimality.

#### **Definition 2**

A point (*x*^∗^,*π*^∗^,*α*^∗^,*β*^∗^,*ε*)is called a *τ*-stationary point of (12) if it satisfies the following relation

19a $$\begin{array}{*{20}l} x^{*}&=\eta_{\omega^{*}\lambda\tau}\left(x^{*}+\tau A^{\top}(y-Ax^{*})\right), \end{array} $$

19b $$\begin{array}{*{20}l} \omega^{*}_{i}&=\frac{\pi^{*}_{i}}{\alpha^{*}}+\frac{1-\pi_{i}^{*}}{\beta^{*}}, \end{array} $$

19c $$\begin{array}{*{20}l} a_{i}^{*}&=\frac{\frac{1-p}{\alpha^{*}}\exp\left(-\frac{\left|x_{i}^{*}\right|}{\alpha^{*}}\right)}{\frac{1-p}{\alpha^{*}} \exp\left(-\frac{\left|x_{i}^{*}\right|}{\alpha^{*}}\right)+\left(\frac{p}{\beta^{*}}\exp\left(-\frac{\left|x_{i}^{*}\right|}{\beta^{*}}\right)\right.} \end{array} $$

19d $$\begin{array}{*{20}l} \pi^{*}&=\sigma_{n-K}(a), \end{array} $$

19e $$\begin{array}{*{20}l} \alpha^{*}&=\sum_{i=1}^{n}\frac{\pi_{i}^{*}\left|x_{i}^{*}\right|+\epsilon}{\sum_{j=1}^{n}\pi_{j}^{*}},\quad \beta^{*}=\sum_{i=1}^{n}\frac{\left(1-\pi_{i}^{*}\right)|x_{i}^{*}|+\epsilon}{\sum_{j=1}^{n}\left(1-\pi_{j}^{*}\right)}. \end{array} $$

#### **Theorem 1**

If (*x*^∗^,*π*^∗^,*α*^∗^,*β*^∗^,*ε*) is a minimizer of (12) then it is a *τ*-stationary point of (12) with $\tau <2\|A\|_{2}^{-2}.$ Viceversa, if (*x*^∗^,*π*^∗^,*α*^∗^,*β*^∗^,*ε*) is a *τ*-stationary point of (12) with $\tau <2\|A\|_{2}^{-2}$, then it is a local minimizer of (12).

The proof can be obtained with similar techniques, devised in [51], and we omit the proof for brevity. This result provides a necessary condition for optimality and shows that, being the function in (12) not convex, *τ*-stationarity points are only local minima. The next theorem ensures that also the sequence (*ζ*^(*t*)^) converges to a limit point which is also a *τ*-stationary point of (12) of the algorithm and, from Theorem 1, a local minimum for (12). Moreover, in Theorem 3, we derive an upper bound on the reconstruction error.

#### **Theorem 2**

(2LMM-ISTA convergence). Let us assume that for every index set *Γ*⊆[*n*]=*K*, the columns of A associated with *Γ* are linearly independent, $\tau ^{(t)} =\tau <2\|A\|_{2}^{-2}$, *u*^(*t*)^=0. Then for any $y\in \mathbb {R}^{m}$, the sequence *ζ*^(*t*)^=(*x*^(*t*)^,*π*^(*t*)^,*α*^(*t*)^,*β*^(*t*)^,*ε*^(*t*)^) generated by Algorithm *1* converges to (*x*^*∞*^,*π*^*∞*^,*α*^*∞*^,*β*^*∞*^,*ε*^*∞*^) which satisfies relations in (19).

#### **Definition 3**

Let A be an *m*×*n* matrix and let 1≤*s*≤*n* be an integer. The matrix *A* is said to satisfy the *s*-restricted isometry property with restricted isometry constant *δ*_*s*_∈(0,1) if, for every *x* with |supp(*x*)|≤*s*, it holds

$$(1-\delta_{s})\|x\|_{{2}}^{2}\leq \|A_{{sx}_{s}}\|_{{2}}^{2}\leq (1+\delta_{s})\|x\|_{{2}}^{2}. $$

#### **Theorem 3**

(2LMM-ISTA: upper bound on the error)Suppose that *A* is an *m*×*n* sampling matrix with restricted isometry constant *δ*_2*K*_. Let *x*^*∞*^ be the output of Algorithm *1* with $\tau ^{(t)} =\tau <2\|A\|_{2}^{-2}$, *u*^(*t*)^=0 and *Λ*^*∞*^=supp(*x*^*∞*^). Let *e*=*x*^*∞*^−*x*_⋆_. If *r*(*x*^*∞*^)_*K*+1_=0 and $\|\sigma _{K}(A^{\top }_{(\Lambda ^{\infty })^{\mathrm {c}}} (y-Ax^{\infty }))\|\leq \|A_{\Lambda ^{\infty }}^{\top }(y-Ax^{\infty })\|=c$, then

$$\begin{array}{*{20}l} \|e_{\Lambda^{\infty}}\|&\leq\frac{1}{1-2\delta_{2K}}\left(\frac{\lambda (1-\delta_{2K})\sqrt{K}}{\beta^{\infty}}+c\delta_{2K}\right) \end{array} $$

and

$$\begin{array}{*{20}l} \left\|e_{(\Lambda^{\infty})^{\mathrm{c}}\cap\Lambda^{\star}}\right\|\leq \frac{c}{1-\delta_{2K}}+\frac{\delta_{2K}}{1-\delta_{2K}}\|e_{\Lambda^{\infty}}\|. \end{array} $$

It should be noticed that the result in Theorem 3 implies that the mean square error $\mathsf {MSE}=\|e\|^{2}/n=\left (c+\frac {\lambda \sqrt {K}}{\beta ^{\infty }}\right)^{2}/n+O(\delta _{2K})$. In this sense, we have provided conditions verifiable a posteriori for convergence in a neighborhood of the solution. This is a common feature in shrinkage-thresholding methods. In [52], it is shown that, in the absence of noise, if certain conditions are satisfied, the error provided by Lasso is *O*(*λ*), where *λ* is the regularization parameter. Since ISTA and FISTA converge to a minimum of the Lasso, we argue that the same estimate holds also for the error between the provided estimations and the true signal.

The proof of Theorems 2 and 3 are postponed to Appendices 3 and 4 and are obtained using arguments of variational analysis and analysis of *τ*-stationary point of (12), respectively.

#### **Example 1**

Computing *δ*_2*K*_ is hard in practice. However, for i.i.d Gaussian and Rademacher matrices, the RIP holds with high probability when *m*≥*c*_0_(*δ*/2)*k* log(*n*e/*k*) where *c*_0_ is a function of isometry constant *δ[*34*]*. To give an example, if *n*=10000, *m*=8000, and *k*=10, then the RIP property holds with probability 0.98 with isometry constant equal to *δ*_2*k*_=0.4. Running the proposed iterative algorithms (ISTA or FISTA) with *λ*=10^−3^, we can empirically check that the condition $\|\sigma _{K}(A^{\top }_{(\Lambda ^{\infty })^{\mathrm {c}}}(y-Ax^{\infty }))\|\leq \|A_{\Lambda ^{\infty }}^{\top }(y-Ax^{\infty })\|\leq c$ is always satisfied with *c*=1.7176·10^−4^. The error of the provided estimate is *MSE*=∥*x*^⋆^−*x*∥^2^/*n*≈1.47·10^−10^ and the estimated upper bound, obtained using Theorem 3, is 5.143·10^−10^. Using error bounds in *[*52*]*, we are able to guarantee that the solutions provided by ISTA and FISTA are accurate with an error only proportional to *λ*=10^−3^.

## Numerical results, experiments, and discussion

In this section, we compare several first-order methods with their versions augmented by the support estimation, in terms of convergence times and empirical probability of reconstruction in the absence and in the presence of noise. It is worth remarking that this does not represent a challenge among all first-order methods for compressed sensing. Our aim is to show that the combination of support detection and estimation using an iterative reweighted first-order method can improve several iterative shrinkage methods. In other terms, we want to show that, given a specific algorithm for CS, the speed and the performance can be improved via its 2-LMM counterpart. Moreover, in order to show that the choice of the weights is important to obtain fast algorithms and good performance, we have employed an iterative shrinkage method for iterative reweighted *ℓ*_1_ minimization algorithm (IRL1). In [45], IRL1 requires to solve at each step a weighted *ℓ*_1_ minimization. This algorithm has computational complexity which is not comparable with the iterative shrinkage/thresholding algorithms since each iteration has complexity of order *O*(*n*^3^). We employ a shrinkage-thresholding method for IRL1 in the spirit of [53] and show that the performance are not as good as in the proposed methods. What we mean as IRL1 is summarized in Algorithm 2.


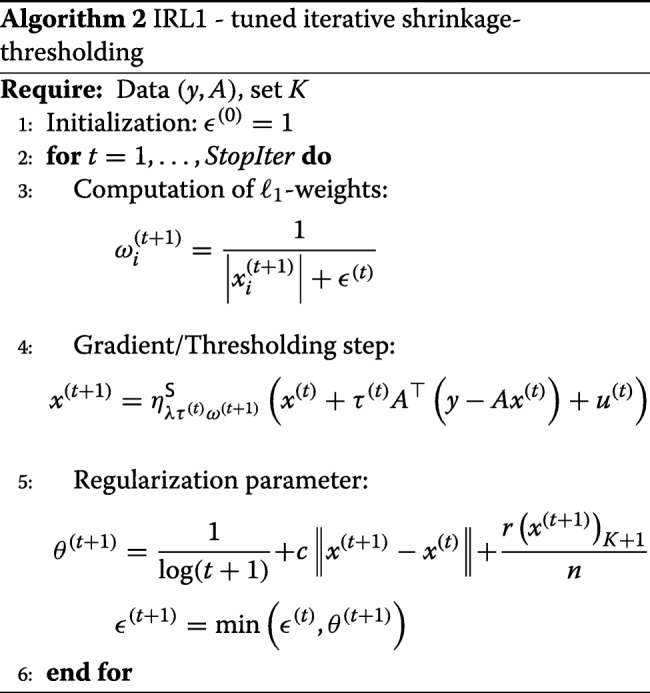


### Reconstruction from noise-free measurements

#### Rate of convergence

As a first experiment, we consider Bernoulli-Gaussian signals [54]. More precisely, the signal to be recovered has length *n*=560 with *k*=50 nonzero elements drawn from a N(0,4), respectively. The sensing matrix *A* with *m*=280 rows is sampled from the Gaussian ensemble with zero mean and variance 1/*m*. We fix *λ*=10^−3^ and *τ*=0.19, and the mixture parameters are initialized , *K*=*k*+10, and *p*=*K*/*n*.

**Fig. 5:**
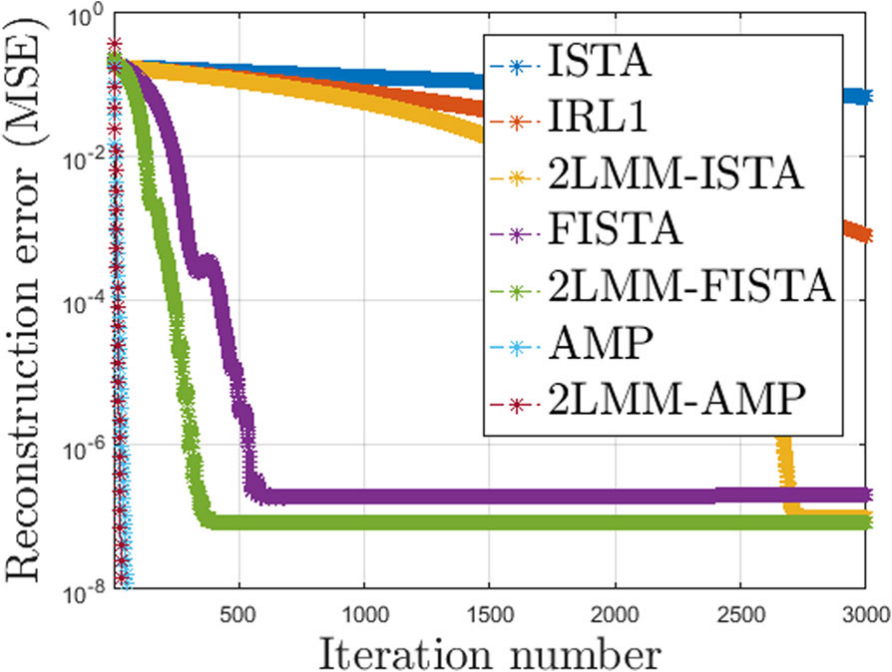
Convergence rate: evolution of the MSE for classical thresholding method algorithms and the corresponding versions with 2-LMM-tuning for sparse Bernoulli-Gaussian signals

In Fig. 5, we compare the convergence rate of ISTA, FISTA, IRL1, and AMP with the corresponding methods with 2-LMM-tuning (2-LMM-ISTA, 2-LMM-FISTA, and 2-LMM-AMP). In particular, the mean square error (MSE) of the iterates *MSE*(*t*)=∥*x*^(*t*+1)^−*x*^⋆^∥^2^/*n* averaged over 50 instances is depicted as a function of the iteration number.

A few comments are in order. The sparsity problem that ISTA, FISTA, and AMP are intended to approximately solve is the same (basis pursuit or Lasso problem). However, the convergence results are different for these iterative algorithms. More precisely, in the absence of noise,

- ISTA and FISTA, under certain conditions, are analytically proved to converge to a minimum of Lasso. This solution is shown to provide only an approximation of the sparse solution *x*^⋆^ which is controlled by Lasso parameter *λ*. More precisely, $\|x^{\star }-\widehat {x}\|_{2}\leq C\lambda $ where $C\in \mathbb {R}$ and perfect reconstruction is not guaranteed.
- AMP instead is not proved to converge in a deterministic sense. In [14], only the average case performance analysis is carried out. The authors exploit the randomness of *A* and instead of calculating the limit of ∥*x*^*t*^−*x*^⋆^∥^2^, they show the convergence in the mean square sense $ \mathbb {E}\|x^{t}-x^{\star }\|^{2}\rightarrow 0$.

In Fig. 5 the accuracy of the solution provided by ISTA, FISTA, and AMP are different. The difference of AMP has been already explained. The difference between ISTA and FISTA is due to the fact that *λ*=0.005 for ISTA (to speed up the algorithm) and *λ*=0.001 for FISTA. As already observed, we are not interested in a challenge among all first-order methods for CS. Our aim is to show that the combination of support detection and estimation using an iterative reweighted first-order method can improve a series of iterative shrinkage methods. More precisely, given a specific algorithm for CS, the speed can be improved via its 2-LMM counterpart.

It should be noted that the proposed algorithms are much faster than classical iterative shrinkage-thresholding methods: there is about a 81,37, and 35% of reduction in the number of iterations needed for the convergence of 2-LMM-ISTA, 2-LMM-FISTA, and 2-LMM-AMP, respectively.

#### Effect of the prior distribution

We now show a second experiment: we fix *n*=512 and take the fraction of the nonzero coefficients fixed to *ρ*=*k*/*n* and we study the effect of the nonzero coefficients distribution on the empirical probability of reconstruction for different values of *k*∈[1,250]. More precisely, $x^{\star }_{i}\sim (1-\rho)\delta _{0}(x^{\star }_{i})+\rho g(x^{\star }_{i})$ where *g* is a probability distribution function and *δ*_0_ is the Dirac delta function. In Table 1, the acronyms of the considered distributions are summarized (see also [55]).

**Fig. 6:**
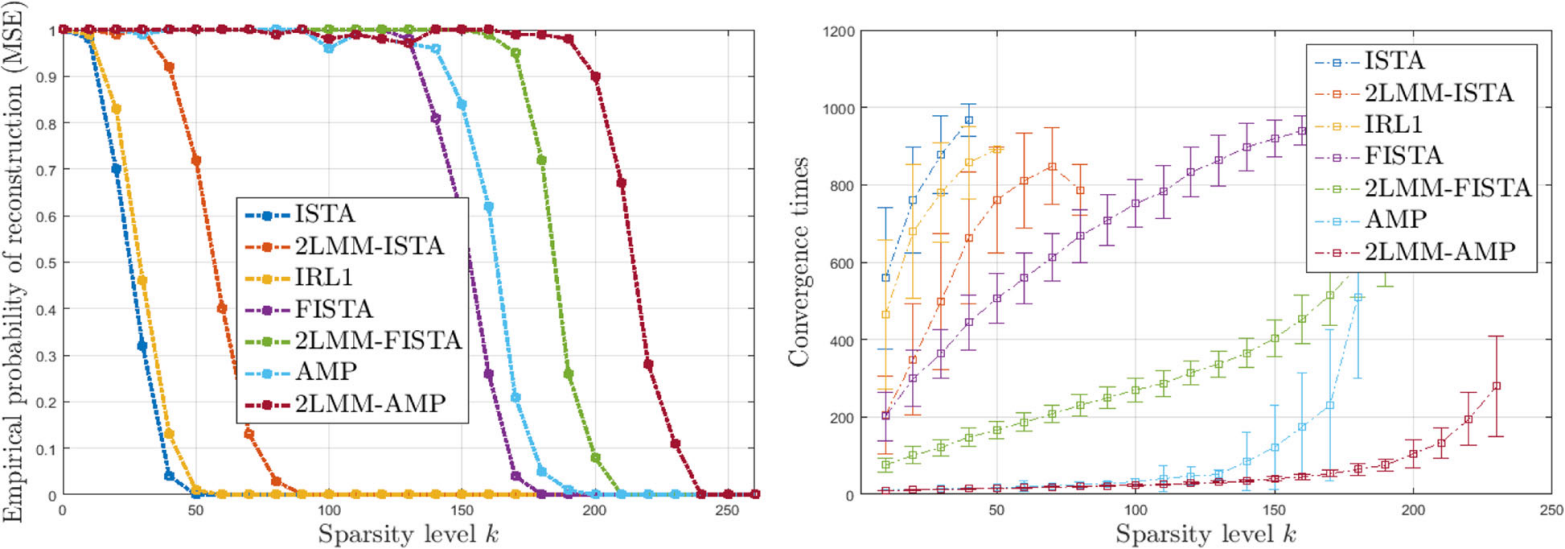
Analysis of performance for L1 signals: empirical probability of successful recovery as a function of the sparsity value *k* with *n*=512 and *m*=350 sparse Bernoulli-Laplace signals (L1)

**Fig. 7:**
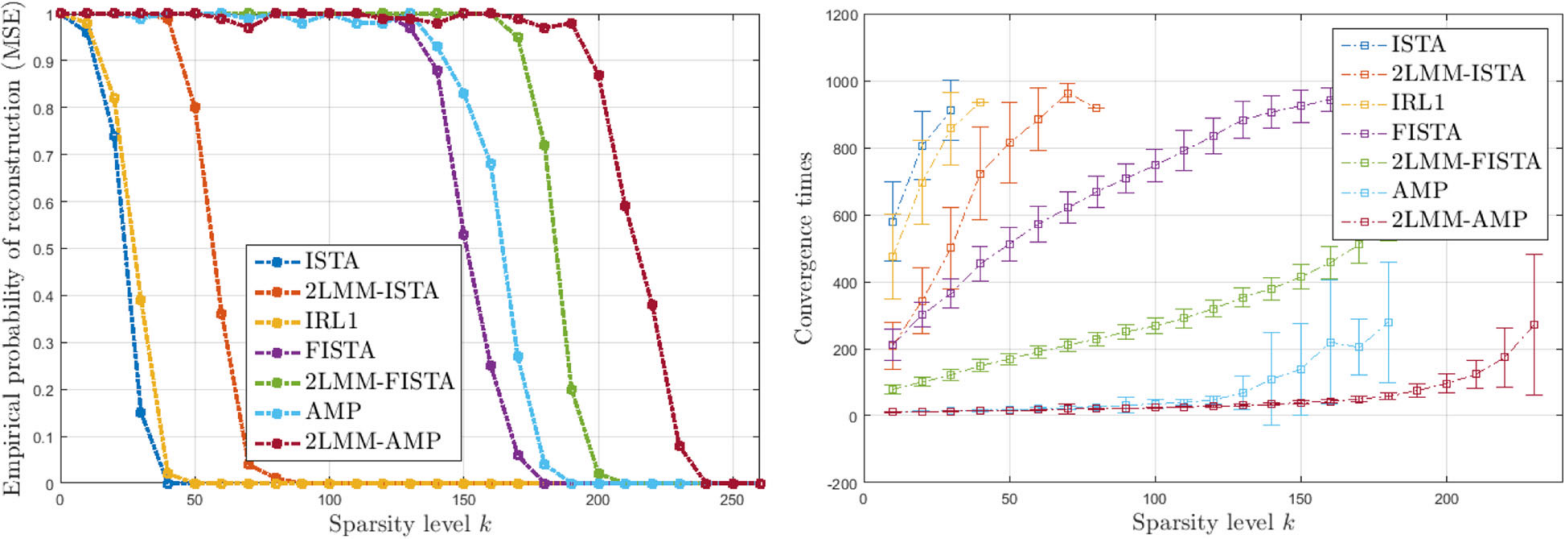
Analysis of performance for G1 signals: empirical probability of successful recovery as a function of the sparsity value *k* with *n*=512 and *m*=350 for sparse Bernoulli-Gaussian signals (G1)

**Fig. 8:**
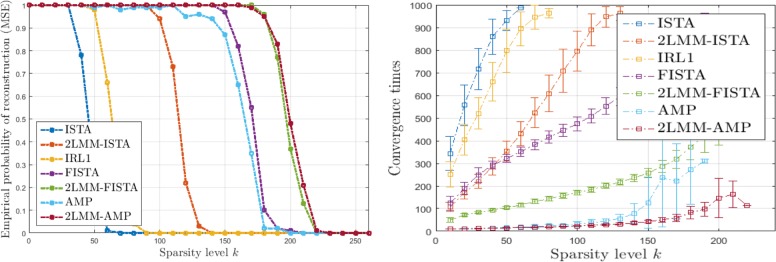
Analysis of performance for U1 signals: empirical probability of successful recovery as a function of the sparsity value *k* with *n*=512 and *m*=350 for sparse Bernoulli-Uniform signals (U1)

**Fig. 9:**
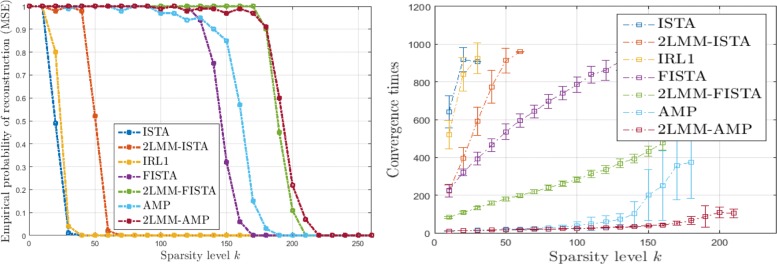
Analysis of performance for 5P1 signals: empirical probability of successful recovery as a function of the sparsity value *k* with *n*=512 and *m*=350 for sparse Bernoulli-Binomial signals (5P1)

Figures 6, 7, 8, and 9 (left) show the empirical recovery success rate, averaged over 50 experiments, as a function of the signal sparsity for different signal priors (see Table 2). For all recovery algorithms, the convergence tolerance has been fixed to 10^−4^. In this case, the elements of matrix *A* with *m*=350 are sampled from a normal distribution with variance 1/*m*. We have fixed a total number of iterations equal to 1000. The algorithm parameters have been initialized as in Table 3.

**Table 2:** Nonzero coefficients distribution

Notation	*g*
L1	*Lap*(0,4)
G1	*N*(0,4)
U1	*U*[0,4]
5P1	$\mathbb {P}(x=-1)=\mathbb {P}(x=1)=0.3$
	$\mathbb {P}(x=-5)=\mathbb {P}(x=5)=0.2$

**Table 3:** Parameters of several shrinkage-thresholding algorithms

	*λ*	*τ* ^( *t*)^	*π* ^(0)^	*α* ^(0)^	*K*
ISTA	0.005	0.2	–	–	–
IRL1	0.005	0.2	–	–	66
FISTA	0.001	0.2	–	–	66
2-LMM-ISTA	0.005	0.2		0.1	66
2-LMM-FISTA	0.001	0.2		0.1	66
AMP	0.9	Eq. (17), *χ*=0.9	–	–	–
2-LMM-AMP	0.9	Eq. (17), *χ*=0.9		0.1	66

It should be noticed that for ISTA, IRL1, and 2-LMM-ISTA, we have chosen *λ*=0.005, instead of *λ*=0.001 as in FISTA and 2-LMM-FISTA, in order to speed up the convergence. The convergence of ISTA and IRL1 are extremely slow, and before 1000 iterations, we get only an approximation with an error of order 10^−3^ for sparsity larger than 50 in most of the cases.

It should be noticed that the 2-LMM-tuning improves the performance of iterative shrinkage-thresholding methods in terms of sparsity-undersampling trade-off. For example for 5P1, it turns out that the signal recovery with 2-LMM-tuning is possible with 30%, 63% sparsity level higher than FISTA, and AMP, respectively.

Figures 6, 7, 8, and 9 (right) show the average running times (CPU times in seconds) of the algorithms computed over the successful experiments, and the error bar represents the standard deviation of uncertainty for different signal priors. These graphs demonstrate the benefit of 2-LMM-tuning for iterative shrinkage/thresholding methods. Not only 2-LMM-tuning shows better performance in the reconstruction but it also runs much faster than traditional methods. Despite the additional per iteration computational cost needed to update the mixture parameters, the gain of the 2-LMM-tuning ranges from 2 to over 6 times, depending on the signal prior. The algorithm efficiency can be attributed to the simple form of the model used as parametric representation of the signal and the improved runtime performance comes from the effective denoising so that fewer iterations are required to converge.

### Reconstruction in imperfect scenarios

In this section, we compare the first-order methods with their versions augmented by the support estimation for recovery of signals in imperfect scenarios where the signal is not exactly sparse or the measurements are noisy.

#### Not exactly sparse signals

**Fig. 10:**
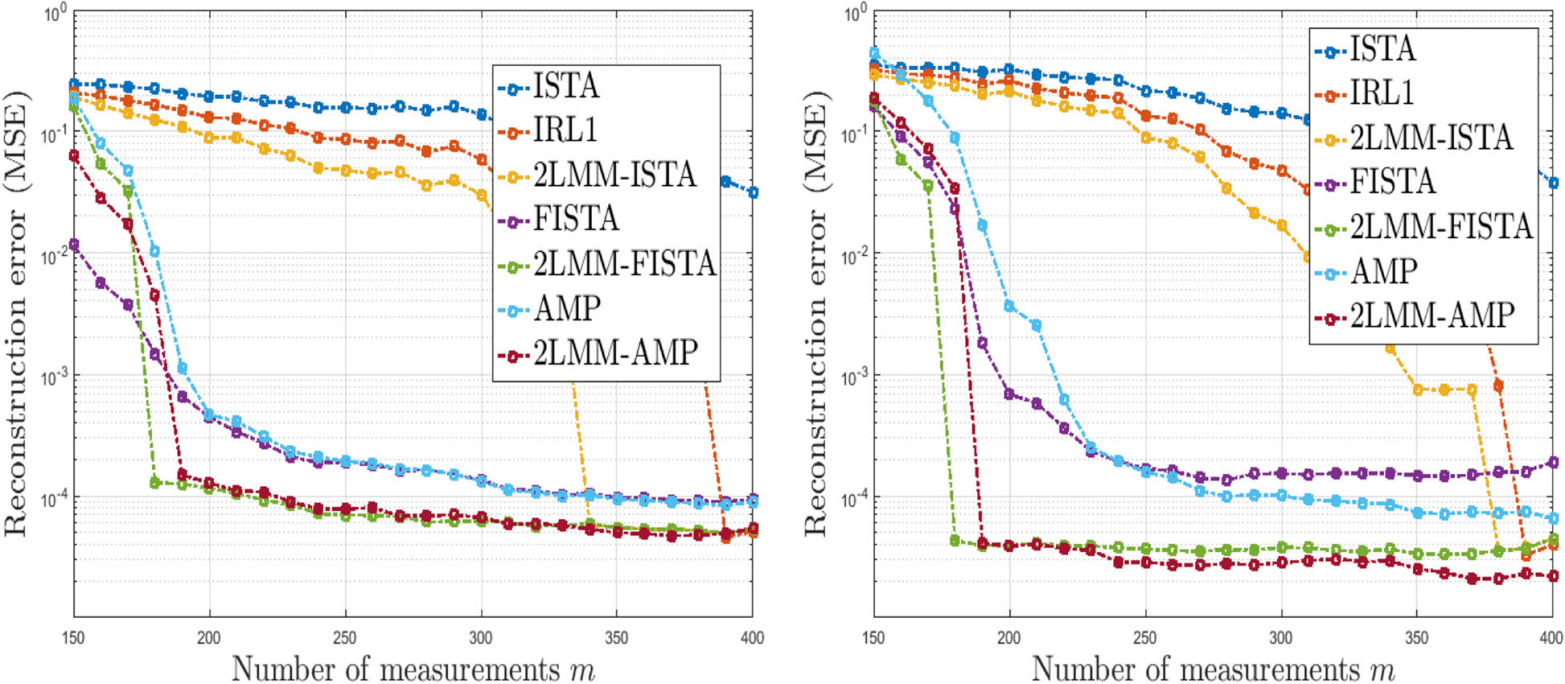
Analysis of performance in imperfect scenarios: MSE versus number of measurements for recovery of not exactly sparse signals (left) and of exactly sparse signals in presence of noise (right), *n*=512, *k*=56

In this experiment, we investigate the performance of first-order methods with 2-LMM-tuning for signals that are not strictly sparse. We consider signals of the form *x*^⋆^=*x*_0_+*ξ* where *x*_0_ is drawn i.i.d from the ensemble of Bernoulli-Gaussian signals (see G1 in Table 2) of length *n*=512 with sparsity level *k*=56 and $\xi \in \mathbb {R}^{n}$ is a vector whose components are distributed as N(0,*σ*^2^) with *σ*=0.01. Here, *k* can be interpreted as the compressibility level of the signal *x*^⋆^. The sensing matrix *A* with *m*∈[160,360] rows is sampled from the Gaussian ensemble with zero mean and variance 1/*m*. Then, the reconstruction is performed using measurements *y*=*Ax*^⋆^. The first-order methods are compared with their versions augmented by 2-LMM-tuning: the parameters have been initialized as follows: , *K*=*k*+10, and *p*=*K*/*n*. Figure 10 shows the MSE achieved by 3000 iterations of the algorithms as a function of measurements used for the reconstruction. As we can see, the algorithms with 2-LMM-tuning have similar reconstruction performance and outperform significantly their traditional counterpart (for example 2-LMM-AMP needs 62% of measurements required by AMP to reach a similar accuracy of MSE=10^−4^). In this case the improved performance comes from the effective denoising so that fewer iterations are required to achieve a better accuracy.

#### Reconstruction with noisy measurements

We now fix *n*=512 and we study the performance of the proposed methods in scenarios with inaccurate measurements according to (1). In this case, *x*^⋆^ is a random Bernoulli-Uniform signal (see U1 in Table 2) with sparsity degree *k*=56 and the noise *η* is white Gaussian noise with standard deviation *σ*=0.01. In this case, the parameters are set as follows: , *K*=*k*+10, and *p*=*K*/*n*. The MSE achieved by 3000 iterations is depicted as a function of the number of measurements used in the reconstruction. Also in this case, the best results are obtained by methods with 2-LMM-tuning. The efficiency of the proposed algorithms allows to reduce the number of measurements required to achieve a satisfactory level of accuracy. As can be noticed from Fig. 10 (right), 2-LMM-FISTA and 2-LMM-AMP need fewer observations (about 180 measurements) than FISTA and AMP (about 270 measurements) to achieve MSE =10^−4^.

**Fig. 11:**
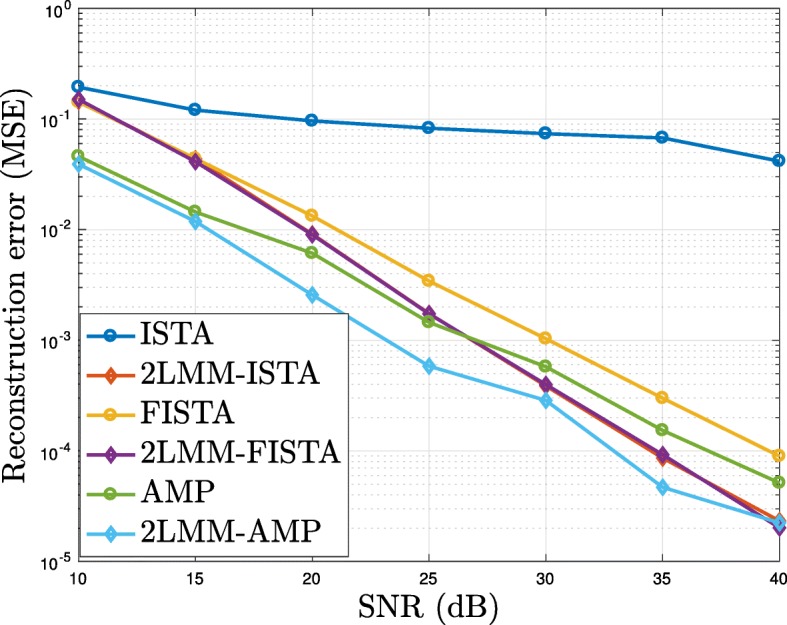
Analysis of robustness against noise: the mean square error is depicted as a function of the SNR

In Fig. 11, we show that the proposed methods are robust against noise. More precisely, the mean square error, averaged over 50 runs, and obtained after 3000 iterations, is depicted as a function of signal-to-noise ratio (SNR), defined as follows

$$\text{SNR}=\frac{\mathbb{E}\left[\|Ax\|^{2}\right]}{m\sigma^{2}}. $$

As to be expected, as the SNR increases the MSE goes to zero. Moreover, the MSE of the proposed algorithms are smaller than those obtained via classical iterative thresholding algorithms. As already observed, the MSE of ISTA is very high compared to the other methods. This is due to the fact that the algorithm is very slow and the number of iterations are not enough to reach a good recovery error.

In our setup, we have considered only Gaussian Noise and the robustness against multiplicative noise is out of our scope. This would require a drastic modification of the proposed algorithms and will be subject of future research. For example, when the underlying sparse or approximately sparse signal is a vector of nonnegative intensities whose measurements are corrupted by Poisson noise, standard CS techniques cannot be applied directly, as the Poisson noise is signal-dependent [56*,*^57^]. In this case, the rationale of our method can be adapted combining the use of mixtures models with exponential distribution instead Laplace distribution with penalized negative Poisson log-likelihood objective function with nonnegativity constraints. We refer to [58] for more details on the model and on the implementations of related iterative thresholding algorithms.

If the multiplicative noise is due to hardware’s amplification and is not signal-dependent, we can model the measurements as follows

$$y=DAx^{\star}+\eta $$

where $D=\text {diag}(\exp (\mathcal {N} (0, \sigma ^{2})))$, where *D* is a diagonal matrix of noise and *σ* is the parameter governing the amplitude of decalibration. To address this problem, the most standard existing approach is the blind calibration for compressed sensing [59]. More precisely, the sparse regularization is exploited considering *A* as an inaccurate estimate of the true measurement system *A*^⋆^=*DA* and $y=Ax^{\star }+(\widehat {A}-A)x^{\star }+\eta \approx Ax^{\star }+\varepsilon +\eta $ with *ε* an estimate of the magnitude of this added noise $\left \|\left (\widehat {A}-A\right)x^{\star }\right \|$. We refer to [59] for more sophisticated approaches of blind supervised calibration and adaptations of classical methods that perform both calibration and reconstruction.

### Comparison with structured sparsity-based Bayesian compressive sensing

Many authors have recently developed structured sparsity-based Bayesian compressive sensing methods in order to deal with different signals arising in several applications and adaptively explore the statistical structure of nonzero pattern. We refer the interested reader to the repository http://people.ee.duke.edu/~lcarin/BCS.html for an introduction to Bayesian compressive sensing (BCS) methods and to structured sparsity-based Bayesian compressive sensing.

For example, [60] proposes a spatio-temporal sparse Bayesian method to recover multichannel signals simultaneously, not only exploiting temporal correlation within each channel signal but also exploiting inter-channel correlations among different signals. This method has been shown to provide several advantages in applications in brain computer interface and electroencephalography-based driver’s drowsiness estimation in terms of measurements for reconstruction and computational load. In [61], using a new bilinear time-frequency representation, a redesigned BCS approach is developed for the problem of spectrum estimation of multiple frequency-hopping signals, arising in various communication and radar applications in the context of multiple-input multiple-output (MIMO) operations in the presence of random missing observations. Another example of structured sparsity-based Bayesian compressive sensing comes from the context of reconstruction of signals and images that are sparse in the wavelet basis [62] or in DCT basis with applications to image compression. More precisely, in [62], the statistical structure of the wavelet coefficients is exploited explicitly using a tree-structured Bayesian compressive sensing approach. This tree structure assumption shows several advantages in terms of number of measurements required for reconstruction.

It is worth remarking that in our approach, we do not use any prior information on the structure of the sparsity pattern and we expect that structured sparsity-based methods outperform our approach. A detailed comparison and an ad hoc adaptation of our approach to all specific frameworks mentioned above is out of the scope of this paper. However, in this section, we propose a numerical comparison of 2-LMM-tuning FISTA with tree-structured wavelet-based Bayesian compressive sensing (WBCS). The implementation of WBCS algorithm used for comparison are implemented via a hierarchical Bayesian framework, with the tree structure incorporated naturally in the prior setting. See TS-BCS for wavelet via MCMC in the repository http://people.ee.duke.edu/~lcarin/BCS.html for a detailed description of the code.

**Fig. 12:**
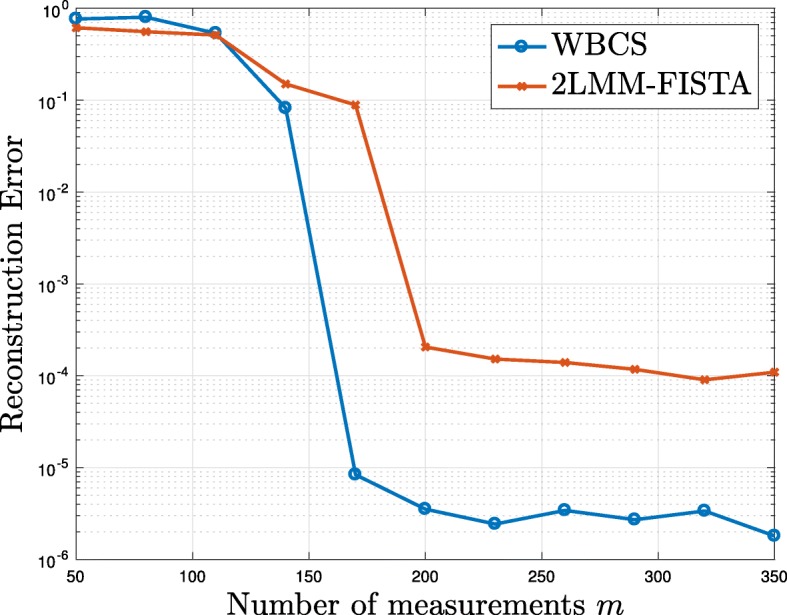
Comparison between WBCS and 2-LMM: reconstruction error of a signal with *n*=512 and *k*=63 as a function of number of measurements

For the comparison, we consider the setting in [62] with a signal of length *n*=512 that are sparse in the Haar wavelet basis and whose coefficients are not independent as in classical compressed sensing framework. Specifically, under the wavelet basis, if a parent node in a wavelet tree is zero or close to zero, with a very large probability, its children nodes are also zero or close to zero. We refer to [62] for details on the generation of the signal. The sparsity of the considered signal is *k*=63. In this case, the parameters are set as follows: , *K*=*k*+10, and *p*=*K*/*n*. In Fig. 12 the reconstruction error achieved by 10000 iterations is depicted as a function of the number of measurements used in the reconstruction. It is worth remarking that WBCS explores the statistical structure of the wavelet coefficients to reduce the number of CS measurements and goes beyond simply assuming that the data are compressible in a wavelet basis. As to be expected, since more a priori knowledge is employed, WBCS shows better performance in terms of reconstruction accuracy. However, the gap is not large and the 2-LMM-FISTA tuning is able to learn the sparsity model and, as soon as the number of measurements is larger than 200, we obtain a good reconstruction accuracy, of order 10^−4^ for 2-LMM-FISTA and of order 10^−5^ for WBCS.

### Deblurring images

In order to show the effectiveness of the 2-LMM-tuning, in this section, we repeat the same experiment proposed in [9] for deblurring two test images (Lena and cameraman). In [9], it has been shown that FISTA significantly outperforms ISTA and other first-order methods in terms of the number of iterations required to achieve a given accuracy. For this reason, we compare the performance of FISTA with our proposed algorithm 2-LMM-FISTA.

**Fig. 13:**
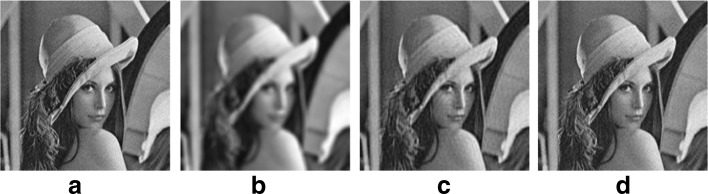
Analysis of performance for Lena image deblurring: **a** original image, **b** acquired image, **c** FISTA reconstruction MSE (dB) = − 10.718 (after 1000 iterations), **d** 2-LMM-FISTA reconstruction MSE (dB) = − 13.3958 (after 1000 iterations)

**Fig. 14:**
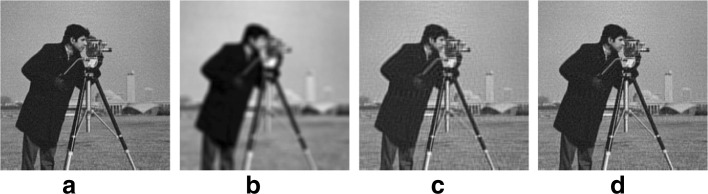
Analysis of performance for cameraman image deblurring: **a** original image, **b** acquired image, **c** FISTA reconstruction MSE (dB) = − 11.4731 (after 1000 iterations), **d** 2-LMM-FISTA reconstruction MSE (dB) = − 14.4731 (after 1000 iterations)

In the considered setting, both images have equal size 256×256 and all pixels of the original images are scaled into the range between 0 and 1. A Gaussian blur of size 9×9 and standard deviation 4 are applied to both images and an additive zero mean white Gaussian noise with standard deviation 10^−4^ is added. The original and observed images are given in Figs. 13 and 14, respectively. We then test FISTA and 2-LMM-FISTA for recovery, where *y* represents the (vectorized) observed image, and *A*=*RW*, where *R* is the matrix representing the blur operator and *W* is the inverse of a three-stage Haar wavelet transform. The regularization parameter is fixed as in [9] *λ*=2·10^−5^, and the blurred image is used as initial condition. For 2-LMM-ITA, the parameters are set as follows: *α*^(0)^=1, , *K*=10000, and *p*=*K*/*n*.

**Fig. 15:**
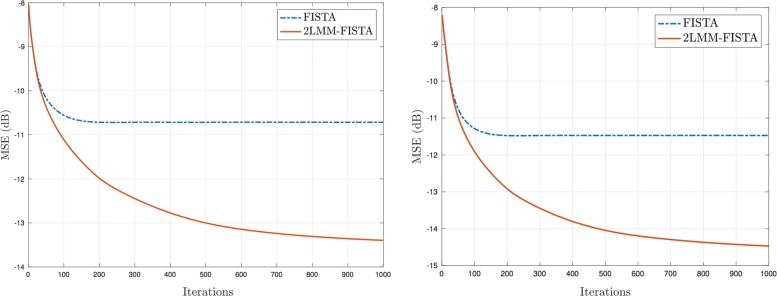
Analysis of rate of convergence in image deblurring: Lena (left) and cameraman (right): MSE (dB) versus number of iterations

In Fig. 15, the evolution of the error in dB is depicted as a function of the number of iterations. In particular, the images produced by 2-LMM-FISTA exhibit better quality than those obtained by using the classical version of FISTA. In Fig. 13 and 14, the reconstructions obtained by the competing methods are shown for Lena and cameraman, respectively. As can be seen in Figs. 13 and 14, 2-LMM-FISTA achieves significantly better visual quality, as the amount of noise is minimized and visual artifacts are greatly reduced. This is also reflected by the reconstruction PSNR, which is significantly higher for 2-LMM-FISTA.

## Conclusions

In this paper, we proposed a new method to perform both support detection and sparse signal estimation in compressed sensing. Combining MAP estimation with the parametric representation of the signal with a Laplace mixture model, we formulated the problem of reconstruction as a reweighted *ℓ*_1_ minimization. Our contribution includes theoretical derivation of necessary and sufficient conditions for reconstruction in the absence of noise. Then, 2-LMM-tuning has been proposed to improve the performance of several iterative shrinkage-thresholding algorithms. Iterative procedures have been designed by combining EM algorithm with classical iterative thresholding methods. Numerical simulations show that these new algorithms are faster than classical ones and outperform them in terms of phase transitions. Topics of our current research is to use similar technique based on Laplace mixture models for robust compressed sensing, where measurements are corrupted by outliers (see [63] and reference therein).

## Appendix 1: proof of Proposition 1

he proof of Proposition 1 is a direct consequence of a more general result on compressible prior result, formally stated in Proposition 1 in [17].

### **Lemma 1**

(Proposition 1.1 in [17]) Suppose $x_{n}\in \mathbb {R}^{n}$ is i.i.d. with respect to a distribution *p*(*x*). Denote *p*(*x*):=0 for *x*<0, and $\overline {p}(x) := p(x)+p(-x)$ for *x*≥0 as the probability density function of |*X*_*n*_|, and $\overline {F}(t) := \mathbb {P}(|X| \leq t)$ as its cumulative density function. Assume that $\overline {F}$ is continuous and strictly increasing on some interval [*a*,*b*], with $\overline {F}(a)=0$ and $\overline {F}(b)=1$, where 0≤*a*≤*b*≤*∞*. For any 0<*κ*≤1, define the following function

$$G_{2}[p](\kappa):=\frac{\int_{0}^{\overline{F}^{-1}(1-\kappa)}x^{2}\overline{p}(x)\mathrm{d}x}{\int_{0}^{\infty}x^{2}\overline{p}(x)\mathrm{d}x}. $$

If $\mathbb {E}[|X|^{q} ]<\infty $ for some *q*∈(0,*∞*). Then, *G*_*q*_[*p*](*κ*) is also well defined for *κ*=0, and for any sequence *k*_*n*_ such that ${\lim }_{n\rightarrow \infty }k_{n}/n=\kappa \in [0,1]$ the following holds almost surely

$${\lim}_{n\rightarrow\infty}\overline{\varrho}_{k_{n}}(x_{n})^{2}=G_{2}[p](x) $$

**Proof of Proposition 1.** For 2-LMM, we have

$${p}(x)=\frac{(1-p)}{2\alpha}\mathrm{e}^{-|x|/\alpha}+\frac{p}{2\beta}\mathrm{e}^{-|x|/\beta}, $$

from which we get

$$\overline{F}(t)=\int_{0}^{t}\overline{p}(x)\mathrm{d}x=1-(1-p)\mathrm{e}^{-t/\alpha}-p\mathrm{e}^{-t/\beta}. $$

Then, let $t=t(\kappa)=\overline {F}^{-1}(1-\kappa)$, i.e., be the solution

$$(1-p)\mathrm{e}^{-t/\alpha}+p\mathrm{e}^{-t/\beta}=\kappa. $$

We now compute

$$\begin{array}{*{20}l} \int_{0}^{t}x^{2}\overline{p}(x)\mathrm{d}x&=(1-p)\left[\alpha^{2}-\left(\alpha^{2}+\alpha t+\frac{t^{2}}{2}\right)\mathrm{e}^{-t/\alpha}\right]\\ &\quad+p\left[\beta^{2}-\left(\beta^{2}+\beta t+\frac{t^{2}}{2}\right)\mathrm{e}^{-t/\beta}\right] \end{array} $$

and

$$\begin{array}{*{20}l} \int_{0}^{\infty} x^{2}\overline{p}(x)\mathrm{d}x&=(1-p)\alpha^{2}+p\beta^{2}. \end{array} $$

Then, the assertion is proved by applying Lemma 1 and we obtain

$${{\begin{aligned} & {\lim}_{n\rightarrow\infty}\overline{\varrho}_{k_{n}}(x_{n})^{2}\\ &=\frac{(1\,-\,p)\left[\alpha^{2}\,-\,\left(\alpha^{2}\,+\,\alpha t\,+\,\frac{t^{2}}{2}\right)\mathrm{e}^{-t/\alpha}\right]\,+\,p\left[\beta^{2}\,-\,\left(\beta^{2}+\beta t\,+\,\frac{t^{2}}{2}\right)\mathrm{e}^{-t/\beta}\right]}{(1\,-\,p)\alpha^{2}\,+\,p\beta^{2}} \end{aligned}}} $$

## Appendix 2: proof of Proposition 2

ecall that

$$\begin{array}{*{20}l} f(x|y;\Theta)&=f(x;\Theta)f(y|x;\Theta)\\ f(x;\Theta)&=\prod_{i=1}^{n}f(x_{i};\Theta)\\ f(x_{i};\Theta)&=\frac{(1-p)}{2\alpha}\mathrm{e}^{-|x_{i}|/\alpha}+\frac{p}{2\beta}\mathrm{e}^{-|x_{i}|/\beta} \end{array} $$

and define

$$\begin{array}{*{20}l} f(x,z;\Theta)&=\prod_{i=1}^{n}f(x_{i},z_{i};\Theta)\\ f(x_{i},z_{i};\Theta)&=\frac{(1-p)z_{i}}{2\alpha}\mathrm{e}^{-|x_{i}|/\alpha}+\frac{p(1-z_{i})}{2\beta}\mathrm{e}^{-|x_{i}|/\beta}\\ f(z|x;\Theta)&=\prod_{i=1}^{n}f(z_{i}|x_{i};\Theta)\\ f(z_{i}|x_{i};\Theta)&=\frac{\frac{(1-p)z_{i}}{2\alpha}\mathrm{e}^{-|x_{i}|/\alpha}+\frac{p(1-z_{i})}{2\beta}\mathrm{e}^{-|x_{i}|/\beta}}{\frac{(1-p)}{2\alpha}\mathrm{e}^{-|x_{i}|/\alpha}+\frac{p}{2\beta}\mathrm{e}^{-|x_{i}|/\beta}} \end{array} $$

### **Lemma 2**

The log-likelihood function defined in (6) is given by

$$\begin{array}{*{20}l} L(x;\Theta)&=\sum_{i=1}^{n}\sum_{z_{i}\in\{0,1\}}f(z_{i}|x_{i};\Theta)\log\left({f(x_{i},z_{i};\Theta)}\right)\\ &\quad-\sum_{i=1}^{n}\sum_{z_{i}\in\{0,1\}}f(z_{i}|x_{i};\Theta)\log\left({f(z_{i}|x_{i};\Theta)}\right)\\ &\quad+\log(f(y|x;\Theta)) \end{array} $$

### *Proof*

We have the following series of equalities:

$$ \begin{aligned} &L(x;\Theta)\,-\,\log(f(y|x;\Theta))\\ &\qquad=\log\left[\prod_{i=1}^{n}f(x_{i};\Theta)\right]=\sum_{i=1}^{n}\log\left[f(x_{i};\Theta)\right]\\ &\qquad=\sum_{i=1}^{n}\log\left(\sum_{z_{i}\in\{0,1\}}f(z_{i}|x_{i};\Theta)\frac{f(x_{i},z_{i};\Theta)}{f(z_{i}|x_{i};\Theta)}\right) \end{aligned} $$

from which we conclude

$$\begin{array}{*{20}l} L(x;\Theta)-\log(f(y|x;\Theta))&\geq\sum_{i=1}^{n}\,\sum_{z_{i}\in\{0,1\}}f(z_{i}|x_{i};\Theta)\\ &\quad\times\left[\log\left(\frac{f(x_{i},z_{i};\Theta)}{f(z_{i}|x_{i};\Theta)}\right)\right] \end{array} $$

where the last inequality follows from Jensen’s inequality ($\mathbb {E}(\phi (x))\leq \phi (\mathbb {E}(x))$ and *ϕ*(*x*)= log(*x*) concave function) and “ *S*_*i*_∼*f*(*z*_*i*_|*x*_*i*_;*Θ*)” subscripts above indicate that the expectations are with respect to *S* drawn from *f*(*z*_*i*_|*x*_*i*_;*Θ*) distribution. By noticing that *f*(*x*_*i*_,*z*_*i*_;*Θ*)/*f*(*z*_*i*_|*x*_*i*_;*Θ*)=*f*(*x*_*i*_|*Θ*), i.e., constant with respect to *z*_*i*_, we notice that the above inequality is actually an equality. The proof is then concluded by using the logarithm properties:

$$\begin{array}{*{20}l} &L(x;\Theta)-\log(f(y|x;\Theta))\\ &\quad=\sum_{i=1}^{n}\sum_{z_{i}\in\{0,1\}}f(z_{i}|x_{i};\Theta)\log\left({f(x_{i},z_{i};\Theta)}\right)\\ &\qquad-\sum_{i=1}^{n}\sum_{z_{i}\in\{0,1\}}f(z_{i}|x_{i};\Theta)\log\left({f(z_{i}|x_{i};\Theta)}\right) \end{array} $$

### **Proposition 4**

Given *y*,*A*,*Θ*,

20 $$ -L(x;\Theta)= \left\{\begin{array}{ll} J(x,\widehat{\pi};\Theta)-\sum_{i=1}^{n}H(\widehat{\pi}_{i}) & \text{if }y= Ax\\ +\infty & \text{if }y\neq Ax \end{array}\right.  $$

where

21 $$\begin{array}{*{20}l}  {J}(x,\widehat{\pi};\Theta)&=\sum_{i=1}^{n}\left[\frac{\widehat{\pi}_{i}|x_{i}|}{\alpha}+{\widehat{\pi}_{i}}\log\alpha-\pi_{i}\log(1-p)\right.\\ &\quad+\left.\frac{(1\,-\,\widehat{\pi}_{i})|x_{i}|}{\beta}\,+\,{(1\,-\,\widehat{\pi}_{i})}\log\beta\,-\,(1\,-\,\widehat{\pi}_{i})\log p\right]\!, \end{array} $$

*H*(*t*)=−*t* log*t*−(1−*t*) log(1−*t*) is the natural entropy function with *t*∈[0,1] and $\widehat {\pi }=\widehat {\pi }_{i}(x_{i})=f(z_{i}=1|x_{i};\Theta)$.

### *Proof*

From Lemma 2 and defining $\widehat {\pi }_{i}=\widehat {\pi }_{i}(x_{i};\Theta)=f(z_{i}=1|x_{i};\Theta)$

$$\begin{array}{*{20}l}  -L(x;\Theta)\!&=\sum_{i=1}^{n}\left[\widehat{\pi}_{i}\frac{|x_{i}|}{\alpha}+\widehat{\pi}_{i}\log\alpha-\widehat{\pi}_{i}\log{(1-p)}\right.\\ &\quad+\!\left.\frac{(1\,-\,\widehat{\pi}_{i})|x_{i}|}{\beta}\,+\,{(1\,-\,\widehat{\pi}_{i})}\log{\beta}\,-\,{(1\,-\,\widehat{\pi}_{i})} \log{p}\right]\\ &\quad+\!n\log 2\,+\,\sum_{i=1}^{n}\!\left(\!-\widehat{\pi}_{i} \log(\widehat{\pi}_{i})\,-\,(1\!-\widehat{\pi}_{i})\!\log(1\!\!-\widehat{\pi}_{i})\!\right)\\ &\quad+\log\delta_{\{y=Ax\}} \end{array} $$

we obtain

$$\begin{array}{*{20}l} L(x;\Theta)=-J(x,\widehat{\pi};\Theta)+\sum_{i=1}^{n}H(\widehat{\pi}_{i})+\log\delta_{\{y=Ax\}} \end{array} $$

which gives (9). □

**Proof of Proposition 2.** Let us consider *J*(*x*,*π*;*Θ*) and minimize with respect to *π*_*i*_ by taking *x* all the other variable fixed. By imposing the constraint

$$\frac{\partial J(x,\pi;\Theta)}{\partial \pi_{i}}-\sum_{i=1}^{n}\frac{\partial H(\pi_{i};\Theta)}{\partial \pi_{i}}=0 $$

we get

$$\begin{array}{*{20}l} \log\frac{1-\pi_{i}}{\pi_{i}}=\frac{|x_{i}|}{\beta}-\frac{|x_{i}|}{\alpha}-\log\left(\frac{\beta}{\alpha}\frac{1-p}{p}\right) \end{array} $$

and the minimizing value is given by

$$\widehat{\pi}_{i}=\frac{1}{1+\mathrm{e}^{-|x_{i}|\left(\frac{1}{\alpha}-\frac{1}{\beta}\right)}\frac{\beta}{\alpha}\frac{1-p}{p}}=f(z_{i}=1|x_{i};\Theta) $$

for which $\frac {\partial ^{2} J(x,\pi ;\Theta)}{\partial \pi _{i}^{2}}(\widehat {\pi }_{i})-\sum _{i=1}^{n}\frac {\partial ^{2} H(\pi _{i};\Theta)}{\partial \pi ^{2}_{i}}\geq 0$. From last equality and from Proposition 4, we conclude the thesis.

## Appendix 3: proof of Theorem 2

n this section, we prove rigorously Theorem 2, which guarantees the convergence of 2-LMM-ISTA to a limit point. We start from the following preliminary results.

Let $V:\mathbb {R}^{n}\times \Sigma _{n-K}\times \mathbb {R}\times \mathbb {R}\times \mathbb {R}\rightarrow \mathbb {R}$ be the function defined in (12)

22 $$ \begin{aligned} & V(x,\pi,\alpha,\beta,\epsilon)\\ & \quad=\frac{1}{2}\|y-Ax\|_{2}^{2} +\lambda J_{\epsilon} (x,\pi;\Theta) - \lambda \sum_{i=1}^{n} H (\pi_{i}) \end{aligned}  $$

where $H:\left [0,1\right ]\rightarrow \mathbb {R}$ is the natural entropy function *H*(*ξ*)=−*ξ* log*ξ*−(1−*ξ*) log(1−*ξ*). and *J*_*ε*_ is defined in (11).

### **Lemma 3**

(Partial minimizations)

$$\begin{array}{*{20}l} \widehat{\pi}&=\widehat{\pi}(x,\alpha,\beta,\epsilon)=\underset{\pi\in{\Sigma_{n-K}}}{\mathrm{arg\ min}} V(x,\pi,\alpha,\beta,\epsilon)\\ \widehat{\alpha}&=\widehat{\alpha}(x,\pi,\beta,\epsilon)=\underset{\alpha>0}{\mathrm{arg\ min}} V(x,\pi,\alpha,\beta,\epsilon)\\ \widehat{\beta}&=\widehat{\beta}(x,\pi,\alpha,\epsilon)=\underset{\beta>0}{\mathrm{arg\ min}} V(x,\pi,\alpha,\beta,\epsilon) \end{array} $$

Then, it holds true that 
 and

$$\widehat{\pi}=\sigma_{n-K}(a) $$

with

$$a_{i}=\frac{\frac{(1-p)}{\alpha}\exp\left(-\frac{|x_{i}|}{\alpha}\right)}{\frac{(1-p)}{\alpha} \exp\left(-\frac{|x_{i}|}{\alpha}\right)+\frac{p}{\beta}\exp\left(-\frac{|x_{i}|}{\beta}\right)} $$

### *Proof*

By differentiating *V*(*x*,*π*,*α*,*β*,*ε*) with respect to *α* and imposing the first-order condition, we obtain

$$\frac{\partial V}{\partial \alpha}=-\frac{\epsilon}{\alpha^{2}}-\frac{\sum_{i=1}^{n}\pi_{i}|x_{i}|}{\alpha^{2}}+\frac{\sum_{i=1}^{n}\pi_{i}}{\alpha}=0 $$

from which

$$\widehat{\alpha}=\frac{\sum_{i=1}^{n}\pi_{i}|x_{i}|+\epsilon}{\sum_{i=1}^{n}\pi_{i}}=\frac{\sum_{i}\pi_{i}|x_{i}|+\epsilon}{\|\pi\|_{1}}. $$

Checking $\frac {\partial ^{2} V}{\partial \alpha ^{2}}(\widehat {\alpha })\geq 0$, we conclude that $\widehat {\alpha }$ is the minimizing value of *V*(*x*,*π*,*α*,*β*,*ε*).

In analogous way, the expression for $\widehat {\beta }$ can be derived.

We now show that $\widehat {\pi }=\sigma _{n-K}(a)$ is the minimizing value of *V*(*x*,*π*,*α*,*β*,*ε*) for fixed *x*,*α*,*β*,*ε*, i.e., $V(x,\widehat {\pi },\alpha,\beta,\epsilon)\leq V(x,{\pi },\alpha,\beta,\epsilon)$ for all *π*∈*Σ*_*n*−*K*_.

Let *a* be the vector satisfying

$$\begin{array}{*{20}l} \frac{\partial V}{\partial \pi_{i}}={|x_{i}|}\left(\frac{1}{\alpha}-\frac{1}{\beta}+\log\frac{\alpha}{\beta}\frac{1-p}{p}\right)-\log\frac{1-\pi_{i}}{\pi_{i}}=0, \end{array} $$

given by

$$a_{i}=\frac{\frac{(1-p)}{\alpha}\exp\left(-\frac{|x_{i}|}{\alpha}\right)} {\frac{(1-p)}{\alpha}\exp\left(-\frac{|x_{i}|}{\alpha}\right)+\frac{p}{\beta}\exp\left(-\frac{|x_{i}|}{\beta}\right)} $$

and define *γ*=− log(1−*r*(*a*)_*n*−*K*+1_) where *r*(*a*) is the nonincreasing rearrangement of *a*. It should be noticed that

23 $$\begin{array}{*{20}l}  \underset{\pi\in\Sigma_{n-K}}{\text{arg\ min}} V(x,\widehat{\pi},\alpha,\beta,\epsilon) = \underset{\pi\in\Sigma_{n-K}}{\text{arg\ min}} V(x,\widehat{\pi},\alpha,\beta,\epsilon)+\gamma\|\pi\|_{0} \end{array} $$

being *γ*∥*π*∥_0_=(*n*−*K*)*γ* just a constant for *π*∈*Σ*_*n*−*K*_.

The minimum of (23) can be calculated by minimizing with respect to each *π*_*i*_ individually and

$$\begin{array}{*{20}l} V(x,\widehat{\pi},\alpha,\beta,\epsilon)+\gamma\|\pi\|_{0}=g(\pi_{i})+C, \end{array} $$

where *C* is independent of *π*_*i*_ and

$$g(\pi_{i})\,=\,\pi_{i}{|x_{i}|}\!\left(\frac{1}{\alpha}\,-\,\frac{1}{\beta}\right) + \pi_{i}\log\left(\frac{\alpha}{\beta}\frac{1-p}{p}\right)-H(\pi_{i})+\gamma\!|\pi_{i}|^{0}. $$

To derive the minimum, we distinguish two cases, *π*_*i*_=0 and *π*_*i*_≠0. In the first case, the element-wise cost is (ignoring the constant terms) 0. In the second case, the minimum cost (again ignoring the constant terms) is attained for *π*_*i*_=*a*_*i*_ if *π*_*i*_≠0. Comparing the cost for both cases, i.e, *g*(*a*_*i*_)<0, we obtain

$$\begin{array}{*{20}l} &g(a_{i})=a_{i}\log\left(\frac{1-a_{i}}{a_{i}}\right)-H(a_{i})+\gamma<0\\ &\log(1- a_{i})<-\gamma\\ &a_{i}>1-\mathrm{e}^{-\gamma} \end{array} $$

By definition of *γ*, we get

$$\underset{\pi_{i}}{\text{arg\ min}}\,{g}(\pi_{i})= \left\{\begin{array}{ll} a_{i} & \text{if }a_{i}>1-\mathrm{e}^{-\gamma}=r(a)_{n-K+1}\\ 0 & \text{otherwise}. \end{array}\right. $$

From this result and the fact that

$$\widehat{\pi}_{i}=\sigma_{n-K}(a)_{i}= \left\{\begin{array}{ll} a_{i} & \text{if }a_{i}\geq r(a)_{n-K+1}\\ 0 & \text{if }a_{i}< r(a)_{n-K+1} \end{array}\right. \in\Sigma_{n-K} $$

we conclude that $\widehat {\pi }$ is the minimizing value of *V* for fixed *x*,*α*,*β*,*ε*. □

### **Lemma 4**

Define the surrogate functional

24 $$  \begin{aligned} V^{\mathcal{S}}(x,a,\pi,\alpha,\beta,\epsilon)& =V(x,\pi,\alpha,\beta,\epsilon)+\frac{1}{2\tau}\|x-a\|_{2}^{2}\\ &\quad-\left.\frac{1}{2}\|A(x-a)\|_{2}^{2}\right], \end{aligned}  $$

then

$$\eta_{\lambda\tau\omega}(a+\tau A^{\top}(y-Aa))=\underset{x\in\mathbb{R}^{n}}{\mathrm{arg\ min}} V^{\mathcal{S}}(x,a,\pi,\alpha,\beta,\epsilon) $$

with $\omega _{i}=\frac {\pi _{i}}{\alpha }+\frac {1-\pi _{i}}{\beta }$.

### *Proof*

By developing the least squares in (12) is straightforward to show that

$$\begin{array}{*{20}l}  V^{\mathcal{S}}(x,a,\pi,\alpha,\beta,\epsilon)&=\frac{1}{2}\|x-(a+\tau A^{\top}(y-Aa))\|_{2}^{2}\\ &\quad+\sum_{i=1}^{n}\omega_{i}|x_{i}|+\chi(y,A,a,\pi,\epsilon,\alpha,\beta) \end{array} $$

where *χ* is a function independent of *x*. By differentiating the function with respect *x*, we obtain the thesis. □

### **Proposition 5**

The function *V* defined in (22) is not increasing along the iterates *ζ*^(*t*)^ = (*x*^(*t*)^,*π*^(*t*)^,*α*^(*t*)^,*β*^(*t*)^,*ε*^(*t*)^).

### *Proof*

From Lemma 3 and 4, it should be noticed that, for each time $t\in \mathbb {N}$, we have

$$\begin{array}{*{20}l} \alpha^{(t+1)}&=\underset{\alpha}{\text{arg\ min}}V\left(x^{(t+1)},\pi^{(t+1)},\alpha,\beta^{(t+1)},{\epsilon^{(t+1)}}\right)\\ \beta^{(t+1)}&=\underset{\beta}{\text{arg\ min}} V\left(x^{(t+1)},\pi^{(t+1)},\alpha^{(t)},\beta,{\epsilon^{(t+1)}}\right). \end{array} $$

Then

$$\begin{array}{*{20}l} V\left(\zeta^{(t+1)}\right)&=V\left(x^{(t+1)},\pi^{(t+1)},\alpha^{(t+1)},\beta^{(t+1)},{\epsilon^{(t+1)}}\right)\\ &\leq V\left(x^{(t+1)},\pi^{(t+1)},\alpha^{(t)},\beta^{(t+1)},{\epsilon^{(t+1)}}\right)\\ &\leq V\left(x^{(t+1)},\pi^{(t+1)},\alpha^{(t)},\beta^{(t)},{\epsilon^{(t+1)}}\right). \end{array} $$

Since *V*(*x*,*π*,*α*,*β*,*ε*) is an increasing function in *ε* and being *ε*^(*t*+1)^= min{*ε*^(*t*)^,*θ*^(*t*+1)^}≤*ε*^(*t*)^ by definition, we obtain

$$\begin{array}{*{20}l} &V\left(x^{(t+1)},\pi^{(t+1)},\alpha^{(t)},\beta^{(t)},{\epsilon^{(t+1)}}\right)\\ &\qquad\leq V\left(x^{(t+1)},\pi^{(t+1)},\alpha^{(t)},\beta^{(t)},{\epsilon^{(t)}}\right) \end{array} $$

and therefore, using

$$\pi^{(t+1)}=\underset{\pi\in\Sigma_{n-K}}{\text{arg\ min}} V\left(x^{(t+1)},\pi,\alpha^{(t)},\beta^{(t)},{\epsilon^{(t+1)}}\right). $$

see Lemma 3, we get

$$\begin{array}{*{20}l} V\left(\zeta^{(t+1)}\right)&\leq V\left(x^{(t+1)},\pi^{(t+1)},\alpha^{(t)},\beta^{(t)},{\epsilon^{(t)}}\right)\\ &\leq V\left(x^{(t+1)},\pi^{(t)},\alpha^{(t)},\beta^{(t)},{\epsilon^{(t)}}\right). \end{array} $$

It should be noticed that for all *x*

$$V^{\mathcal{S}}(x,x,\pi,\alpha,\beta,{\epsilon})=V(x,\pi,\alpha,\beta,{\epsilon}) $$

and

$$V^{\mathcal{S}}(x,a,\pi,\alpha,\beta,{\epsilon})\geq V(x,x,\pi,\alpha,\beta,{\epsilon}) $$

for all *a*≠*x*. Then, we have

$$\begin{array}{*{20}l} V\left(\zeta^{(t+1)}\right)&\leq V\left(x^{(t+1)},\pi^{(t)},\alpha^{(t)},\beta^{(t)},{\epsilon^{(t)}}\right)\\ &= V^{\mathcal{S}}\left(x^{(t+1)},x^{(t+1)},\pi^{(t)},\alpha^{(t)},\beta^{(t)},{\epsilon^{(t)}}\right)\\ &\leq V^{\mathcal{S}}\left(x^{(t+1)},x^{(t)},\pi^{(t)},\alpha^{(t)},\beta^{(t)},{\epsilon^{(t)}}\right)\\ &\leq V^{\mathcal{S}}\left(x^{(t)},x^{(t)},\pi^{(t)},\alpha^{(t)},\beta^{(t)},{\epsilon^{(t)}}\right)\\ &\leq V\left(x^{(t)},\pi^{(t)},\alpha^{(t)},\beta^{(t)},{\epsilon^{(t)}}\right)\leq V\left(\zeta^{(t)}\right). \end{array} $$

The following lemma implies that these algorithms converge numerically when the number of iterations goes to infinity.

### **Lemma 5**

Let (*x*^(*t*)^) be the sequence generated by 2-LMM-ISTA, then *x*^(*t*+1)^−*x*^(*t*)^→0 as *t*→*∞*.

### *Proof*

If *α*^(*t*)^→0 or *β*^(*t*)^→0 as *t*→*∞*, we have *ε*^(*t*)^→0 and, by definition of *ε*^(*t*)^= min{*ε*^(*t*−1)^,*θ*^(*t*)^} and $\theta ^{(t)}=\frac {1}{\log (t+1)}+c\|x^{(t)}-x^{(t-1)}\|+r(x^{(t)})_{K+1}/n$, we get

$$ {\lim}_{t\rightarrow\infty}c\left\|x^{(t+1)}-x^{(t)}\right\|_{2}=0 $$

and the assertion is true.

If instead neither *α*^(*t*)^ nor *β*^(*t*)^ converge to zero, then there exists a constant *τ*>0 and a sequence of integers $\{T_{\ell }\}:\mathbb {N}\rightarrow \mathbb {N}$ such that *T*_*ℓ*_→*∞*, as *ℓ*→*∞* and $\min \left \{\alpha ^{(T_{\ell })},\beta ^{(T_{\ell })}\right \} > \chi $ for all $\ell \in \mathbb {N}$. It holds true in general that from Proposition 5, we have

25 $$\begin{array}{*{20}l} &\lambda\left[\sum_{i=1}^{n}{\pi_{i}^{(t)}}\log\alpha^{(t)}+\sum_{i=1}^{n}{\left(1-\pi_{i}^{(t)}\right)}\log\beta^{(t)}-n\log 2\right]\\ &\quad\leq\frac{1}{2}\left\|y-Ax^{(t)}\right\|_{2}^{2}+\lambda\left[\sum_{i=1}^{n}{\pi_{i}^{(t)}}\log\alpha^{(t)}\right.\\ &\qquad+\sum_{i=1}^{n}{\left(1-\pi_{i}^{(t)}\right)}\log\beta^{(t)}-\sum_{i=1}^{n}H(\pi_{i})\\ &\qquad-\left.\sum_{i=1}^{n}{\pi_{i}^{(t)}}\log (1-p)-\sum_{i=1}^{n}{(1-\pi_{i}^{(t)})}\log p\right]\\ &\quad\leq V\left(x^{(t)},\pi^{(t)},\alpha^{(t)},\beta^{(t)},{\epsilon^{(t)}}\right)\\ &\quad\leq V\left(x^{(1)},\pi^{(1)},\alpha^{(1)},\beta^{(1)},{\epsilon^{(1)}}\right). \end{array} $$

Then, we have that also for the subsequence *T*_*ℓ*_ it holds

26 $$\begin{array}{*{20}l} & V\left(x^{(T_{\ell})},\pi^{(T_{\ell})},\alpha^{(T_{\ell})},\beta^{(T_{\ell})},{\epsilon^{(T_{\ell})}}\right)\\ &\geq\!\lambda\!\left[\!\sum_{i=1}^{n}{\pi_{i}^{(T_{\ell})}}\!\log\alpha^{(T_{\ell})}\,+\,\sum_{i=1}^{n}\!{\left(1\,-\,\pi_{i}^{(T_{\ell})}\right)} \!\log\beta^{(T_{\ell})}\,-\,n\log 2\right] \\ &\geq\lambda(n\log\chi-n\log2). \end{array} $$

Since $\tau <\|A\|^{-2}_{2}$, we have

27 $$ \begin{aligned} 0&\leq\frac{1}{2\tau}\left(1-\tau\|A\|^{2}\right)\left\|x^{(t)}-x^{(t+1)}\right\|^{2}\\ &\leq\frac{1}{2\tau}\left(x^{(t)}-x^{(t+1)}\right)^{\top}\left(I-\tau A^{\top}A\right)\left(x^{(t)}-x^{(t+1)}\right)\\ &=V^{\mathcal{S}}\left(x^{(t+1)},x^{(t)},\pi^{(t)},\alpha^{(t)},\beta^{(t)},{\epsilon^{(t)}}\right)\\ &\quad-V\left(x^{(t+1)},\pi^{(t)},\alpha^{(t)},\beta^{(t)},{\epsilon^{(t)}}\right)\\ \end{aligned}  $$

Consider the following sum for all $\ell \in \mathbb {N}$

$$\begin{array}{*{20}l}  0&\leq\sum_{t=1}^{T_{\ell}}\frac{1}{2\tau}\left(x^{(t)}\!-x^{(t+1)}\right)^{\top}\left(I-\tau A^{\top}A\right)\left(x^{(t)}-x^{(t+1)}\right)\\ &\overset{(a)}{=}\sum_{t=1}^{T_{\ell}}\left[V^{\mathcal{S}}\left(x^{(t+1)},x^{(t)},\pi^{(t)},\alpha^{(t)},\beta^{(t)},{\epsilon^{(t)}}\right)\right.\\ &\quad-\left.V\left(x^{(t+1)},\pi^{(t)},\alpha^{(t)},\beta^{(t)},{\epsilon^{(t)}}\right)\right]\\ &\overset{(b)}{\leq}\sum_{t=1}^{T_{\ell}}\left[V^{\mathcal{S}}\left(x^{(t)},x^{(t)},\pi^{(t)},\alpha^{(t)},\beta^{(t)},{\epsilon^{(t)}}\right)\right.\\ &\quad-\left.V\left(x^{(t+1)},\pi^{(t)},\alpha^{(t)},\beta^{(t)},{\epsilon^{(t)}}\right)\right]\\ &\overset{(c)}{\leq}\sum_{t=1}^{T_{\ell}}\left[V\left(x^{(t)},\pi^{(t)},\alpha^{(t)},\beta^{(t)},{\epsilon^{(t)}}\right)\right.\\ &\quad-\left.V\left(x^{(t+1)},\pi^{(t+1)},\alpha^{(t)},\beta^{(t)},{\epsilon^{(t)}}\right)\right]\\ &\overset{(d)}{\leq}\sum_{t=1}^{T_{\ell}}\left[V\left(x^{(t)},\pi^{(t)},\alpha^{(t)},\beta^{(t)},{\epsilon^{(t)}}\right)\right.\\ &\quad-\left.V\left(x^{(t+1)},\pi^{(t+1)},\alpha^{(t)},\beta^{(t)},{\epsilon^{(t+1)}}\right)\right]\\ &\overset{(e)}{\leq}\sum_{t=1}^{T_{\ell}}\left[V\left(x^{(t)},\pi^{(t)},\alpha^{(t)},\beta^{(t)},{\epsilon^{(t)}}\right)\right.\\ &\quad-\left.V\left(x^{(t+1)},\pi^{(t+1)},\alpha^{(t+1)},\beta^{(t+1)},{\epsilon^{(t+1)}}\right)\right]\\ &\overset{(f)}{=}V\left(x^{(1)},\pi^{(1)},\alpha^{(1)},\beta^{(1)},{\epsilon^{(1)}}\right)\\ &\quad-V\left(x^{(T_{\ell}+1)},\pi^{(T_{\ell}+1)},\alpha^{(T_{\ell}+1)},\beta^{(T_{\ell}+1)},{\epsilon^{(T_{\ell}+1)}}\right) \\ &\overset{(g)}{\leq} V\left(x^{(1)},\pi^{(1)},\alpha^{(1)},\beta^{(1)},{\epsilon^{(1)}}\right)-\lambda(n\log\chi-n\log 2)\\ &=C' \end{array} $$

where

- Follows from (25)
- Follows from the fact that
  $$x^{(t+1)}=\underset{x\in\mathbb{R}^{n}}{\text{arg\ min}} V^{\mathcal{S}}\left(x,x^{(t)},\pi^{(t)},\alpha^{(t)},\beta^{(t)},{\epsilon^{(t)}}\right) $$
  (see Lemma 4)
- Is a consequence of the following relations
  $$\begin{array}{*{20}l}  V^{\mathcal{S}}\!\left(x^{(t)}\!,x^{(t)}\!,\pi^{(t)}\!,\alpha^{(t)}\!,\beta^{(t)}\!,{\epsilon^{(t)}}\!\right)\! =\!V\!\left(x^{(t)},\pi^{(t)},\alpha^{(t)},\beta^{(t)},{\epsilon^{(t)}}\right)\end{array} $$
  and
  $$\pi^{(t+1)}=\underset{\pi\in\Sigma_{n-K}}{\text{arg\ min}} V\left(x^{(t+1)},\pi,\alpha^{(t)},\beta^{(t)},{\epsilon^{(t)}}\right) $$
  (see Lemma 3);
- and (e) Are following from
  $$\begin{aligned} \alpha^{(t+1)}&=\underset{\alpha}{\text{arg\ min}} V\left(x^{(t+1)},\pi^{(t+1)},\alpha,\beta^{(t)},{\epsilon^{(t)}}\right), \\ \beta^{(t+1)}&=\underset{\beta}{\text{arg\ min}} V\left(x^{(t+1)},\pi^{(t+1)},\alpha^{(t+1)},\beta,{\epsilon^{(t)}}\right) \end{aligned} $$
  (see Lemma 3) and from the fact that *V*(*x*,*π*,*α*,*β*,*ε*) is an increasing function in *ε* and *ε*^(*t*+1)^≤*ε*^(*t*)^ by definition.

We conclude that for all $\ell \in \mathbb {N}$

$$ \sum_{t=1}^{T_{\ell}}\frac{1}{2\tau}\left(x^{(t)}-x^{(t+1)}\right)^{\top}\left(I-\tau A^{\top}A\right)\left(x^{(t)}-x^{(t+1)}\right)\leq C'.$$

By letting *ℓ*→*∞*, we obtain that the series is convergent and, we obtain the necessary condition

$$\begin{array}{*{20}l}  0\leq\frac{1}{2\tau}\left(x^{(t)}-x^{(t+1)}\right)^{\top}\left(I-\tau A^{\top}A\right)\left(x^{(t)}-x^{(t+1)}\right)\rightarrow0 \end{array} $$

as *t*→*∞* and from inequality in (27) the assertion is proved.

(f) Follows by noticing that the series is telescopic
(g) Is a direct consequence bound in (26)

□

### **Lemma 6**

The sequence $(x^{(t)})_{t\in \mathbb {N}}$ is bounded

### *Proof*

We now prove that both *α*^(*t*)^ and *β*^(*t*)^ must be upper bounded. From (25), there exists a constant

$$C=\frac{V\left(x^{(1)},\pi^{(1)},\alpha^{(1)},\beta^{(1)},{\epsilon^{(1)}}\right)}{\lambda}+n\log 2 $$

such that

28 $$  \sum_{i=1}^{n}{\left(1-\pi_{i}^{(t)}\right)}\log\beta^{(t)}+\sum_{i=1}^{n}{\pi_{i}^{(t)}}\log\alpha^{(t)}\leq C.  $$

Suppose ad absurdum that *β*^(*t*)^ is unbounded (similar consideration can be done if *α*^()^ is unbounded), then there exists a sequence $t_{\ell }:\mathbb {N}\rightarrow \mathbb {N}$ such that $\beta ^{(t_{\ell })}\rightarrow \infty $ as *ℓ*→*∞*. By (28), we have $\alpha ^{(t_{\ell })}\rightarrow 0$. In fact, since $\phantom {\dot {i}\!}\pi ^{t_{\ell }}\in \Sigma _{n-K}$ and $\phantom {\dot {i}\!}\beta ^{(t_{\ell })}>1$ definitively (by unboundeness), we have

$$\begin{array}{*{20}l} &K\log\beta^{(t_{\ell})}+\sum_{i\in\text{supp}\left(\pi^{(t_{\ell})}\right)}\pi_{i}^{(t_{\ell})}\log\alpha^{(t_{\ell})}\\ &\quad\leq\sum_{i=1}^{n}{\left(1-\pi_{i}^{(t)}\right)}\log\beta^{(t)}+\sum_{i=1}^{n}{\pi_{i}^{(t)}}\log\alpha^{(t)}\\ &\quad\leq C \end{array} $$

and the inequality is satisfied if and only if $\alpha ^{(t_{\ell })}\rightarrow 0$. Consequently, by definition of $\phantom {\dot {i}\!}\alpha ^{(t_{\ell })}$, also $\phantom {\dot {i}\!}\epsilon ^{(t_{\ell })}\rightarrow 0$ and

29 $$ r\left(x^{(t^{\ell})}\right)_{K+1}\rightarrow0,  $$

where we recall that $r(x^{(t^{\ell })})$ is the nonincreasing rearrangement of $\phantom {\dot {i}\!}x^{(t_{\ell })}$. Let *Δ*:={*i*∈[*n*]:∃*ε*>0 and $(t_{\ell })_{q\in \mathbb {N}}$ for which $|x_{i}^{(t_{\ell })}|>\epsilon \}$, then from (29), we have |*Δ*|≤*K*. Since |*x*_*i*_|>|*x*_*j*_| for all *i*∈*Δ* and *j*∈*Δ*^c^, then

$$\begin{array}{*{20}l} a^{(t_{\ell})}_{i}&=\left({1+\frac{p}{1-p} \frac{\alpha^{(t_{\ell}-1)}}{\beta^{(t_{\ell_{1}}-1)}} \mathrm{e}^{|x_{i}^{(t_{\ell})}|\left(\frac{1}{\alpha^{(t_{\ell}-1)}}-\frac{1}{\beta^{(t_{\ell}-1)}}\right)}}\right)^{-1}\\ &\geq a^{(t_{\ell})}_{j} \end{array} $$

The application of hard thresholding yields $\pi ^{(t_{\ell })}=\sigma _{n-K}\left (a^{(t_{\ell })}\right),$ and we have $\pi _{i}^{(t_{\ell })}=0$ and $\omega _{i}^{(t_{\ell }+1)}={1}/{{\beta ^{(t_{\ell })}}} \overset {q\rightarrow \infty }{\longrightarrow }0$ for all *i*∈*Δ*.

If *i*∈*Δ*^c^, then $|x_{i}^{t_{\ell }}|\rightarrow 0$ as *ℓ*→*∞* and, consequently, from Lemma 5 also $|x_{i}^{t_{\ell }+1}|\rightarrow 0$ as *ℓ*→*∞*.

Let now *Λ*=supp(*x*^⋆^). We thus have

$$\begin{array}{*{20}l}  \left\|x^{(t_{\ell}+1)}-x^{\star}\right\|^{2}_{2}&=\left\|x^{(t_{\ell}+1)}_{\Delta}-x^{\star}_{\Delta}\right\|^{2}_{2}+ \left\|x^{(t_{\ell}+1)}_{\Delta^{\mathrm{c}}}-x^{\star}_{\Delta^{\mathrm{c}}}\right\|^{2}_{2}\\ &\leq\left\|\left(I-\tau A_{\Delta}^{\top}A\right)\left(x^{(t_{\ell})}-x^{\star}\right)\right\|_{2}^{2}\\ &\quad+{K}\lambda \tau\max_{i\in \Delta}{\omega^{(t_{\ell+1})}_{i}}+\left\|x^{(t_{\ell}+1)}_{\Delta^{\mathrm{c}}}-x^{\star}_{\Delta^{\mathrm{c}}}\right\|^{2}_{2}. \end{array} $$

where the last inequality follows from the triangular inequality.

$$\begin{array}{*{20}l}  \left\|x^{(t_{\ell}+1)}-x^{\star}\right\|^{2}_{2}&\leq\left\|I-\tau A_{\Delta}^{\top}A_{\Delta}\right\|_{2}^{2}\left\|x^{(t_{\ell})}_{\Delta}-x^{\star}_{\Delta}\right\|_{2}^{2}\\ &\quad +\left\|\tau A_{\Delta}^{\top}A_{\Delta^{\mathrm{c}}}\right\|_{2}^{2}\left\|x^{(t_{\ell})}_{\Delta^{\mathrm{c}}}-x^{\star}_{\Delta^{\mathrm{c}}}\right\|_{2}^{2}\\ &\quad+K\lambda \tau\max_{i\in \Delta}{\omega^{(t_{\ell+1})}_{i}}+\left\|x^{(t_{\ell}+1)}_{\Delta^{\mathrm{c}}}-x^{\star}_{\Delta^{\mathrm{c}}}\right\|^{2}_{2}. \end{array} $$

Since |*Δ*|≤*K* and the columns of *A* associated with *Δ* are linearly independent, then the matrix $A_{\Delta }^{\top }A_{\Delta }$ is nonsingular and $\left \|I-\tau A_{\Delta }^{\top }A_{\Delta }\right \|_{2}^{2}=\gamma <1$. As terms $\|x^{(t_{\ell })}_{\Delta ^{\mathrm {c}}}-x^{\star }_{\Delta ^{\mathrm {c}}}\|_{2}^{2}$, $\|x^{(t_{\ell }+1)}_{\Delta ^{\mathrm {c}}}-x^{\star }_{\Delta ^{\mathrm {c}}}\|_{2}^{2}$, and $\max _{i\in \Delta }{\omega ^{(t_{\ell +1})}_{i}}$ are going to zero when *ℓ*→*∞*, there exists $t_{0}\in \mathbb {N}$ and a constant $\chi \in \mathbb {R}$ such that if *t*>*t*_0_ then

$$\begin{array}{*{20}l} \left\|x^{(t_{\ell}+1)}-x^{\star}\right\|_{2}&\leq\gamma \left\|x^{(t_{\ell})}-x^{\star}\right\|_{2}+\chi \end{array} $$

Iterating the argument and letting *ℓ*→*∞*,

$${\lim}_{\ell\rightarrow\infty}\left\|x^{(t_{\ell}+1)}-x^{\star}\right\|_{2}\leq \frac{\chi}{1-\gamma} $$

and we conclude that the sequence $(x^{(t_{\ell })})_{\ell \in \mathbb {N}}$ is bounded and so is $\phantom {\dot {i}\!}(\beta ^{(t_{\ell })})$ from which we get the contradiction. We conclude that $(\alpha ^{(t)})_{t\in \mathbb {N}}$ and $(\beta ^{(t)})_{t\in \mathbb {N}}$ are both upper bounded by a constant *χ*>0 and so $(x^{(t)})_{t\in \mathbb {N}}$:

$$0\leq2\epsilon^{(t)}+\left\|x^{(t)}\right\|_{1}=\sum_{i=1}^{n}{\pi_{i}\alpha^{(t)}}+\sum_{i=1}^{n}{(1-\pi_{i})\beta^{(t)}}\leq \chi n $$

□

### **Proposition 6**

Any accumulation point is a *τ*-stationary point of (12) of the algorithm and satisfies the equalities in (19a)-(19e).

### *Proof*

Suppose that (*x*^*♯*^,*π*^*♯*^,*α*^*♯*^,*β*^*♯*^) is an accumulation point of the sequence $\left ({x}^{(t)},{\pi }^{(t)},{\alpha }^{(t)},{\beta }^{(t)}\right)_{t\in \mathbb {N}}$. Then, there exists a subsequence $\left ({x}^{(t_{\ell })},{\pi }^{(t_{\ell })},{\alpha }^{(t_{\ell })},{\beta }^{(t_{\ell })}\right)_{\ell \in \mathbb {N}}$ that converges to (*x*^*♯*^,*π*^*♯*^,*α*^*♯*^,*β*^*♯*^) as *ℓ*→*∞*. We now show (19d) and we let the reader verify the other conditions by continuity. Since ${\lim }_{\ell \rightarrow \infty }{\pi }^{(t_{\ell })}=\pi ^{\sharp }$, then there exists *ℓ*_0_ such that, ∀*ℓ*>*ℓ*_0_ and ∀*i*∈supp(*π*^*♯*^), $\pi _{i}^{(t_{\ell })}\neq 0$ and

$$\pi_{i}^{(t_{\ell})}=a_{i}^{(t_{\ell})}\overset{\ell\rightarrow\infty}{\rightarrow} \frac{{\exp\left(-\frac{|x^{\sharp}|}{\alpha^{\sharp}}\right)}\frac{1-p}{\alpha^{\sharp}}}{\frac{1-p}{\alpha^{\sharp}} {\exp\left(-\frac{|x^{\sharp}|}{\alpha^{\sharp}}\right)} + \frac{p}{\beta^{\sharp}}{\exp\left(-\frac{|x^{\sharp}|}{\beta^{\sharp}}\right)}}= \pi_{i}^{\sharp}. $$

If *i*∉supp(*π*^*♯*^) we distinguish the following cases.(a) There exists a sequence $\left ({\ell _{q}}\right)_{q\in \mathbb {N}}$ such that $0\neq \pi _{i}^{\big (t_{\ell _{q}}\big)}=a_{i}^{\big (t_{\ell _{q}}\big)}\rightarrow \pi _{i}^{\sharp }=0$. This means that there exists $q_{0}\in \mathbb {N}$ such that ∀*q*>*q*_0_ we have $ a_{i}^{(t_{\ell _{q}})}<a_{j}^{\big (t_{\ell _{q}}\big)},\ \forall j\in \text {supp}\left (\pi ^{\sharp }\right) $ and, by letting *q*→*∞*,

30 $$ \begin{aligned} &\frac{{\exp\left(-\frac{|x_{i}^{\sharp}|}{\alpha^{\sharp}}\right)}\frac{1-p}{\alpha^{\sharp}}}{\frac{1-p}{\alpha^{\sharp}} {\exp\left(-\frac{|x_{i}^{\sharp}|}{\alpha^{\sharp}}\right)}+\frac{p}{\beta^{\sharp}}{\exp\left(-\frac{|x_{i}^{\sharp}|}{\beta^{\sharp}}\right)}}\\ &\qquad\leq\frac{{\exp\left(-\frac{|x_{j}^{\sharp}|}{\alpha^{\sharp}}\right)}\frac{1-p}{\alpha^{\sharp}}}{\frac{1-p}{\alpha^{\sharp}} {\exp\left(-\frac{|x_{j}^{\sharp}|} {\alpha^{\sharp}}\right)}+\frac{p}{\beta^{\sharp}}{\exp\left(-\frac{|x_{j}^{\sharp}|}{\beta^{\sharp}}\right)}} \end{aligned}  $$

(b) There exists ${\ell _{0}}\in \mathbb {N}$ such that $\forall \ell >\ell _{0}\ \pi _{i}^{(t_{\ell })}=0$, from which $ a_{i}^{(t_{\ell })}<a_{j}^{(t_{\ell })},\ \forall j\in \text {supp}\left (\pi ^{\sharp }\right) $ and by letting *ℓ*→*∞* we get (30).

We conclude that for all *i*∉supp(*π*^*∞*^)

$$ \pi^{\sharp}_{i}=0=\sigma_{n-K}\left(\frac{{\exp\left(-\frac{|x^{\sharp}|}{\alpha^{\sharp}}\right)} \frac{1-p}{\alpha^{\sharp}}} {\frac{1-p}{\alpha^{\sharp}}{\exp\left(-\frac{|x^{\sharp}|}{\alpha^{\sharp}}\right)} + \frac{p}{\beta^{\sharp}}{\exp\left(-\frac{|x^{\sharp}|}{\beta^{\sharp}}\right)}}\right)_{i}. $$

□

**Proof of Theorem 2:** From Lemma 6 the sequence (*x*^(*t*)^) is bounded and by the Bolzano Weierstrass Theorem, there exists a subsequence $\left (x^{t_{j}}\right)_{j\in \mathbb {N}}$ such that

31 $$  {\lim}_{j\rightarrow\infty}x^{(t_{j})}={x}^{\infty}  $$

$ {\lim }_{j\rightarrow \infty }\epsilon ^{(t_{j})}={\epsilon }^{\infty } $, $ {\lim }_{j\rightarrow \infty }\alpha ^{(t_{j})}={\alpha }^{\infty } $, $ {\lim }_{j\rightarrow \infty }\beta ^{(t_{j})}={\beta }^{\infty } $, and $ {\lim }_{j\rightarrow \infty }\pi ^{(t_{j})}={\pi }^{\infty } $. We thus have

$$\begin{aligned} {\lim}_{j\rightarrow\infty}\!\left\|x^{(t_{j}+1)}\,-\,{x}^{\infty}\right\|&\leq {\lim}_{j\rightarrow\infty}\!\left\|x^{(t_{j}+1)}\,-\,x^{(t_{j})}\,+\,x^{(t_{j})}\,-\,x^{(t_{j})}\,-\,{x}^{\infty}\right\|\\ &\leq{\lim}_{j\rightarrow\infty}\left\|x^{(t_{j}+1)}-x^{(t_{j})}\right\|+\left\|x^{(t_{j})}-{x}^{\infty}\right\|\\ &=0 \end{aligned} $$

where the second inequality follows from triangular inequality and the last equality follows from Lemma 5 and (31). Since $ {\lim }_{t\rightarrow \infty }x^{(t_{j}+1)}={\lim }_{t\rightarrow \infty }x^{(t_{j})}={x}^{\infty } $, by continuity, we get ${\lim }_{t\rightarrow \infty }a^{(t_{j}+1)}={\lim }_{t\rightarrow \infty }a^{(t_{j})}=a^{\infty }$. This implies that $S:=\text {supp}\left (\pi ^{(t_{j}+1)}\right)=\text {supp}\left (\pi ^{(t_{j})}\right)$ definitely and we deduce that

$${\lim}_{j\rightarrow\infty}\pi^{(t_{j}+1)}_{i}=0={\lim}_{j\rightarrow\infty}\pi^{(t_{j})}_{i}=\pi_{i}^{\infty},\forall i\notin S $$

and

$${\lim}_{j\rightarrow\infty}\pi^{(t_{j}+1)}_{i}={\lim}_{j\rightarrow\infty}a^{(t_{j}+1)}_{i}={\lim}_{j\rightarrow\infty}a^{(t_{j})}_{i}=\pi_{i}^{\infty},\forall i\in S.$$

Moreover

$${\lim}_{j\rightarrow\infty}\epsilon^{(t_{j+1})}={\lim}_{j\rightarrow\infty}\epsilon^{(t_{j})}=\epsilon^{\infty} $$

(*ε*^(*t*)^ is positive and monotonic). By continuity also ${\alpha ^{(t_{j}+1)}\rightarrow \alpha ^{\infty }}$ and ${\beta ^{(t_{j}+1)}\rightarrow \beta ^{\infty }}$ as *j*→*∞*. We conclude by induction that the sequence generated by Algorithm 1 converges to (*x*^*∞*^,*π*^*∞*^,*α*^*∞*^,*β*^*∞*^), which is also a *τ*-stationary point of (12) by Proposition 6.

## Appendix 4: Proof of Theorem 3:

rom Theorem 2, Algorithm 1 converges to (*x*^*∞*^,*π*^*∞*^, *α*^*∞*^,*β*^*∞*^) which is also a *τ*-stationary point of (12). Let *Λ*^*∞*^=supp(*x*^*∞*^) and *k*^*∞*^=|*Λ*^*∞*^|≤*K* by assumption *r*(*x*^*∞*^)_*K*+1_=0. We thus have

$$\begin{array}{*{20}l} a_{i}^{\infty}:&=\frac{1}{1+\frac{\alpha^{\infty}}{\beta^{\infty}}\frac{p}{1-p}\exp\left({|x_{i}^{\infty}|}\left(\frac{1}{\alpha^{\infty}}-\frac{1}{\beta^{\infty}}\right)\right)}\\ &\quad\left\{\begin{array}{ll} =\frac{1}{1+\frac{\alpha^{\infty}}{\beta^{\infty}}\frac{p}{1-p}}&\text{for }i\in(\Lambda^{\infty})^{c}\\ > \frac{1}{1+\frac{\alpha^{\infty}}{\beta^{\infty}}\frac{p}{1-p}}&\text{for }i\in(\Lambda^{\infty}) \end{array}\right. \end{array} $$

Since *k*^*∞*^≤*K* then from (19d)

$$\pi_{i}^{\infty}=\sigma_{n-K}(a^{\infty})_{i}= \left\{\begin{array}{ll} \frac{1}{1+\frac{\alpha^{\infty}}{\beta^{\infty}}\frac{p}{1-p}}&\text{for }i\in T\\ 0&\text{for }i\in T^{c} \end{array}\right. $$

for some *T*⊇*Λ*^*∞*^ with |*T*|=*K*. We deduce from (19e) that

$$\alpha^{\infty}=\frac{\sum_{i\in\Lambda^{\infty}}\pi_{i}^{\infty}|x_{i}|}{\sum_{i\in[n]}\pi_{i}}=0 $$

and $\pi _{i}^{\infty }=1$ for all *i*∈*T* and

$$\beta^{\infty}=\frac{\sum_{i\in\Lambda^{\infty}}(1-\pi_{i}^{\infty})|x_{i}|}{\sum_{i\in[n]}(1-\pi_{i}^{\infty})}=\frac{\|x\|_{1}}{K}. $$

Let *e*:=*x*^⋆^−*x*^*∞*^. Since *x*^*∞*^ is a *τ*-stationary point, it should be noticed that

32 $$\begin{array}{*{20}l} \|e_{\Lambda^{\infty}}\|&\leq\left\|\left(A^{\top}_{\Lambda^{\infty}}A_{\Lambda^{\infty}}\right)^{-1}\right\|\left\|\frac{\lambda}{\beta^{\infty}} \text{sgn}(x^{\infty}_{\Lambda^{\infty}})\right\|\\ &+\left\|\left(A^{\top}_{\Lambda^{\infty}}A_{\Lambda^{\infty}}\right)^{-1}\right\| \left\|A^{\top}_{\Lambda^{\infty}}A_{(\Lambda^{\infty})^{\mathrm{c}}}e_{(\Lambda^{\infty})^{\mathrm{c}}}\right\|\\ &\leq\left\|\left(A^{\top}_{\Lambda^{\infty}}A_{\Lambda^{\infty}}\right)^{-1}\right\|\frac{\lambda \sqrt{K}}{\beta^{\infty}}\\ &+\left\|A^{\top}_{\Lambda^{\infty}}A_{(\Lambda^{\infty})^{\mathrm{c}}\cap\Lambda^{\star}}\right\| \left\|e_{(\Lambda^{\infty})^{\mathrm{c}}\cap\Lambda^{\star}}\right\|\\ &\leq\frac{1}{1-\delta_{2K}}\left(\frac{\lambda \sqrt{K}}{\beta^{\infty}}+{\delta_{2K}}\left\|e_{(\Lambda^{\infty})^{\mathrm{c}}\cap\Lambda^{\star}}\right\|\right). \end{array} $$

We have

33 $$\begin{array}{*{20}l} \left\|A_{(\Lambda^{\infty})^{\mathrm{c}}\cap\Lambda^{\star}}^{\top}(y-Ax)\right\|&\leq\left\|\sigma_{K} \left(A_{(\Lambda^{\infty})^{\mathrm{c}}}^{\top}(y-Ax)\right)\right\|\\ &\leq\left\|A^{\top}_{\Lambda^{\infty}}(y-Ax)\right\|=c \end{array} $$

where the last inequality follows from hypothesis. Moreover, from the triangular inequality

34 $$\begin{array}{*{20}l} &\left\|A_{(\Lambda^{\infty})^{\mathrm{c}}\cap\Lambda^{\star}}^{\top}(y-Ax)\right\|\\ &\qquad\geq\left\|A_{(\Lambda^{\infty})^{\mathrm{c}}\cap\Lambda^{\star}}^{\top}A_{(\Lambda^{\infty})^{\mathrm{c}}\cap\Lambda^{\star}}e_{(\Lambda^{\infty})^{\mathrm{c}}\cap\Lambda^{\star}}\right\|\\ &\qquad-\left\|A_{(\Lambda^{\infty})^{\mathrm{c}}\cap\Lambda^{\star}}^{\top}A_{\Lambda^{\infty}}e_{\Lambda^{\infty}}\right\| \\ &\qquad\geq(1-\delta_{2K})\left\|e_{(\Lambda^{\infty})^{\mathrm{c}}\cap\Lambda^{\star}}\right\|-\delta_{2K}\left\|e_{\Lambda^{\infty}}\right\|  \end{array} $$

Combining (33) and (34), we get

$$\left\|e_{(\Lambda^{\infty})^{\mathrm{c}}\cap\Lambda^{\star}}\right\|\leq \frac{c}{1-\delta_{2K}}+\frac{\delta_{2K}}{1-\delta_{2K}}\left\|e_{\Lambda^{\infty}}\right\|. $$

Using (34) and (32), we obtain

$$\begin{array}{*{20}l} \|e_{\Lambda^{\infty}}\|&\leq\frac{1}{1-2\delta_{2K}}\left(\frac{\lambda(1-\delta_{2K}) \sqrt{K}}{\beta^{\infty}}+c\delta_{2K}\right). \end{array} $$

## Abbreviations

2-LMM: Two-component Laplace mixture model
AMP: Approximate message passing
BCS: Bayesian compressive sensing
BP: Basis pursuit
CS: Compressed sensing
DCT: Discrete cosine transform
IRL1: Iterative reweighting *ℓ*_1_ minimization
ISTA: Iterative shrinkage-thresholding algorithm
FISTA: Fast iterative shrinkage-thresholding algorithm
MAP: Maximum a posteriori
MCMC: Markov chain Monte Carlo
MSE: Mean square error
TS-BCS: Tree-structure Bayesian compressive sensing
WBCS: Wavelet-based Bayesian compressive sensing
